# Characterization of Brazilian spring wheat germplasm and its potential for increasing wheat genetic diversity in Canada

**DOI:** 10.3389/fgene.2023.1125940

**Published:** 2023-03-17

**Authors:** Silvia Barcellos Rosa, Gavin Humphreys, Linda Langille, Harvey Voldeng, Maria Antonia Henriquez, Andrew James Burt, Harpinder Singh Randhawa, Tom Fetch, Colin W. Hiebert, Barbara Blackwell, Taye Zegeye, Allan Cummiskey, Eric Fortier, Pedro Luiz Scheeren, Camila Turra, Brent McCallum

**Affiliations:** ^1^ Centre de recherche sur les grains (CÉROM), Saint-Mathieu-de-Beloeil, QC, Canada; ^2^ Ottawa Research and Development Centre, Agriculture and Agri-Food Canada, Ottawa, ON, Canada; ^3^ Morden Research and Development Centre, Agriculture and Agri-Food Canada, Morden, MB, Canada; ^4^ Lethbridge Research and Development Centre, Agriculture and Agri-Food Canada, Lethbridge, AB, Canada; ^5^ Brandon Research and Development Centre, Agriculture and Agri-Food Canada, Brandon, MB, Canada; ^6^ Charlottetown Research and Development Center, Agriculture and Agri-Food Canada, Charlottetown, PEI, Canada; ^7^ Empresa Brasileira de Pesquisa Agropecuaria (EMBRAPA) Trigo, Passo Fundo, Brazil; ^8^ OR Melhoramento de Sementes, Passo Fundo, Brazil

**Keywords:** wheat, *Triticum aestivum*, breeding, germplasm, genetic diversity, rust, Fusarium

## Abstract

In the present era of climate instability, Canadian wheat production has been frequently affected by abiotic stresses and by dynamic populations of pathogens and pests that are more virulent and aggressive over time. Genetic diversity is fundamental to guarantee sustainable and improved wheat production. In the past, the genetics of Brazilian cultivars, such as Frontana, have been studied by Canadian researchers and consequently, Brazilian germplasm has been used to breed Canadian wheat cultivars. The objective of this study was to characterize a collection of Brazilian germplasm under Canadian growing conditions, including the reaction of the Brazilian germplasm to Canadian isolates/pathogens and to predict the presence of certain genes in an effort to increase genetic diversity, improve genetic gain and resilience of Canadian wheat. Over 100 Brazilian hard red spring wheat cultivars released from 1986 to 2016 were evaluated for their agronomic performance in eastern Canada. Some cultivars showed good adaptability, with several cultivars being superior or statistically equal to the highest yielding Canadian checks. Several Brazilian cultivars had excellent resistance to leaf rust, even though only a few of these tested positive for the presence of either *Lr34* or *Lr16*, two of the most common resistance genes in Canadian wheat. Resistance for stem rust, stripe rust and powdery mildew was variable among the Brazilian cultivars. However, many Brazilian cultivars had high levels of resistance to Canadian and African - Ug99 strains of stem rust. Many Brazilian cultivars had good Fusarium head blight (FHB) resistance, which appears to be derived from Frontana. In contrast FHB resistance in Canadian wheat is largely based on the Chinese variety, Sumai-3. The Brazilian germplasm is a valuable source of semi-dwarf (*Rht*) genes, and 75% of the Brazilian collection possessed *Rht-B1b.* Many cultivars in the Brazilian collection were found to be genetically distinct from Canadian wheat, making them a valuable resource to increase the disease resistance and genetic variability in Canada and elsewhere.

## 1 Introduction

Wheat is one of the primary crops in Canada with approximately 10 Mha seeded annually and a total annual production ranging from approximately 22 Mt to over 37 Mt between 2010 and 2022 (Statistics Canada). Approximately 75% of Canadian wheat production is exported and it is valued worldwide for its excellent quality, and versatility in end-use applications. Canadian wheat cultivars are grouped into marketing classes based on their functional characteristics, growth habit (spring or winter) and geographical origin (eastern or western Canada). The main classes of wheat grown in Canada are Canada Western Red Spring (60% of total wheat grown), Canada Western Amber Durum, Canada Prairie Spring Red and Canada Eastern Soft Red Winter (Cereals Canada, 2023).

Wheat in Brazil is a secondary crop. Approximately 2 Mha of wheat are seeded annually with production reaching 6.3Mt in 2020 (FAOSTAT). However, the Brazilian wheat crop is important for food security with nearly all of the production consumed domestically. Wheat production in Brazil increased 3.7 times from 2000 to 2022, although the cultivated area only increased two-fold (FAOSTAT). Brazil produces only hard red spring wheat, which is classified based on flour characteristics. Fusarium head blight (FHB) and pre-harvest sprouting are important limitations to the cultivation of durum and white soft wheats. The wheat is grown mostly in southern Brazil in the autumn-winter months and harvested in the early summer. Climatic conditions during the growing season are very favourable to the development of fungal diseases. The pathogens survive during the summer on alternative hosts, and the green-bridge leads to high disease pressure. Consequently, superior disease resistance is critical for wheat cultivation in Brazil and good resistance to various diseases has been developed through years of breeding.

Targets for improving wheat in both countries include grain yield, disease resistance, adaptation to climate change and abiotic stresses, and end-use quality. Some of the disease problems are common to both countries, which include FHB and leaf rust.

Collaboration between Brazilian and Canadian breeders and pathologists has existed for decades. Wheat varieties from Brazil, such as Frontana, have been used to breed Canadian wheat cultivars in the past. The genetic studies to characterize the leaf rust resistance of Frontana, carried out in Canada (Dyck et al., 1966; Dyck, 1987; McCallum et al., 2016), identified the resistance genes: *Lr13* and *Lr34*. The identification of new resistance genes provides an opportunity to improve Canadian wheat disease resistance and resilience through genetics derived from Brazilian germplasm. Similarly, Brazilian breeding programs could use Canadian germplasm to improve end-use quality, for example,.

The project started as there was a need for Brazilian breeding programs to characterize their current germplasm for stem rust resistance. The disease had been absent in Brazil for decades, so the resistance of modern cultivars was unknown. While it was known that the stem rust resistance of older cultivars was derived from *Sr31* and *Sr24* (Barcellos, *pers. comm.*), the genetics of more recently released cultivars had not been determined. With the threat of the African stem rust race Ug99 and its variants (Fetch et al., 2021), which rendered *Sr31* and *Sr24* ineffective, it was important to characterize the Brazilian germplasm for stem rust resistance to prevent future losses. Due to the valuable genetic diversity of Brazilian germplasm, a thorough characterization of the collection, in which older cultivars were also included, was undertaken. The ultimate objective was to evaluate the agronomic performance and disease resistance of a comprehensive collection of Brazilian wheat cultivars under Canadian growing conditions and pathogen populations. The analysis used representative Canadian check cultivars for grain yield and other agronomic characteristics along with resistance to FHB, leaf rust, stem rust, stripe rust, and powdery mildew. A secondary objective was to predict the presence or absence of some critical wheat genes in this germplasm collection using molecular markers diagnostic for the presence of these genes.

## 2 Materials and methods

### 2.1 Germplasm and agronomic performance

The Brazilian wheat germplasm evaluated in this study was composed of 111 cultivars organized in two collections (Supplementary Table S1). Collection “A” was composed of cultivars registered in Brazil from 1986 to 2012, while the registration of the collection “B” cultivars dated from 1999 to 2016. Sixteen cultivars were present in both collections. The cultivars were derived from multiple breeding programs in Brazil. Some cultivars were removed during the study because of lack of seed. The Canadian checks were chosen based on disease reaction as controls in nurseries; thus, they do not represent the predominant cultivars in Canada.

Two agronomic trials were conducted in Ottawa (Ontario) and Saint-Mathieu-de-Beloeil (Quebec) in 2017 and 2018 using a randomized complete block design with two replications (4.77 m^2^ plots) without fungicide treatments. The agronomic characteristic evaluated were grain yield (kg/ha), thousand kernel weight (TKW, g), test weight (TW, kg/hL), days to heading (Julian date), days to maturity (Julian date), plant height (cm), and lodging (1–9). Percent grain protein content was measured using GrainSpec NIR machine (FossElectric, United Kingdom).

### 2.2 Leaf rust

Artificially inoculated and irrigated nurseries were used to determine the field leaf rust reaction of the collections. Test entries were seeded in 1 m rows, with three replications each year. *Puccinia triticina* (Eriks.) urediniospores were used to inoculate susceptible spreader rows, regularly spaced between test rows. The inoculum was generated from a representative mixture of the virulence phenotypes found in Canada during the annual national virulence survey in the previous year (McCallum et al., 2021). After purification, characterization and multiplication of *Puccinia triticina* isolates, urediniospores were suspended in light mineral oil (Soltrol, Chevron Phillips Chemical Co.) and sprayed on the leaves of the spreader rows at early tillering. Subsequently, leaf rust developed on the spreader rows and urediniospores were windblown onto the test materials. Leaf rust severity (proportion of the flag leaf infected with leaf rust, %) was rated near maturity at the point of maximum infection using the modified Cobb scale (Peterson e*t al.,* 1948). The entire collection was tested for leaf rust resistance in nurseries at Morden in 2017 and 2018, collection ‘B’ was also evaluated at Morden in 2016.

To determine seedling resistance, each cultivar (five to six plants) was grown indoors to the two leaf stage and inoculated with urediniospores of single purified *P. triticina* isolates, as described by McCallum *et al.* (2021). The isolates used were 96-12-3 MBDS, 128-1 MBRJ, 74-2 MGBJ, 11-180-1 TDBG, 06-1-1 TDBG, and 77-2 TJBJ. Plants were rated to determine the infection type 12–14 days post-inoculation as described in McCallum *et al.* (2021).

### 2.3 Stem rust

The reaction of the Brazilian cultivars to *Puccinia graminis* Pers. f. sp. *Tritici* Eriks. and E. Henn. (*Pgt*) was tested both in the field and greenhouse. In the greenhouse, the collection was tested for reaction to Canadian races QCC, TPM, RHT, TMR, RKQ, QTH, MCC, QFC, and the African Ug99 race TTKSK, as described by Fetch et al. (2021). At 14 days post-inoculation, seedlings were scored for infection type (IT) using a 0–4 scale (Stakman and Levine, 1922), where ITs from 0 to 2 were deemed resistant and 3-4 were considered susceptible. All tests with Ug99 TTKSK were conducted in a Plant Pest Containment Level 3 (PPC3).

For the Morden field trials, entries were planted in 1 m rows with ranges of six rows flanked by susceptible spreader rows. The spreader rows were inoculated with a mixture of Pgt races (TPMKC, TMRTF, RKQSC, RHTSF, QTHJF, RTHJC and MCCFC with AAFC-MRDC isolate numbers 1373, 1,311, 1,312, 1,562, 1,347, 1,561, and 1,541 respectively; from the AAFC—Morden Research and Development Centre (MRDC) Pgt collection of isolates found in Canadian fields) at the jointing stage by spraying urediniospores suspended in a light mineral oil (Soltrol^®^170 Isoparaffin) on a day preceding anticipated overnight dew. Spores from heavily infected spreader rows caused infection of the experimental plots. The populations were rated for stem rust severity and infection response once the susceptible checks showed heavy disease (severities near 80%) which was approximately at anthesis. The modified Cobb scale was used to assess disease severity (Peterson et al., 1948), while infection response: resistant (R), moderately resistant (MR), moderately susceptible (MS), susceptible (S) was assessed using the scale of Roelfs et al. (1992).

### 2.4 Stripe rust

Stripe rust resistance was evaluated in field trials in Lethbridge, AB (2015—collection “A” and 2017) and Creston, BC (2017). A randomized complete block design with two replicates was used in Lethbridge in 2017, while there was no replication at the other sites. Spreaders for stripe rust were composed of a mixture of susceptible cultivars Morocco, SWS18 and AC Barrie. All trials relied on natural infection from the prevalent *Puccinia striiformis* f. sp. *tritici* (*Pst*) populations. Plants were scored for stripe rust infection at wheat anthesis, when the susceptible check lines showed over 50% rust infection. Stripe rust severity was recorded on a 0%–100% severity infection scale.

### 2.5 Powdery mildew

Under natural disease pressure, powdery mildew resistance was evaluated in Charlottetown (PEI) in 2017 and 2018. The trials were conducted using a randomized complete block design with two replicates. The entries were planted in a single 1 m-long rows. A 0 (none) to 9 (completely covered) scale was used to score the disease severity.

### 2.6 Fusarium head blight

The Brazilian collection was screened in inoculated FHB nurseries at Morden (MB) in 2017 and 2018 and Ottawa (ON) in 2021 using a randomized complete block design with three replicates. Plots consisted of a single 1 m row.

In Morden, *Fusarium graminearum* (*Fg*) corn kernel inoculum was prepared using four *Fg* isolates from the Henriquez Spring Wheat (HSW) collection: HSW-15–39 [3-acetyldeoxynivalenol (ADON) chemotype], HSW-15-87 (3-ADON), HSW-15–27 (15-ADON) and HSW-15–57 (15-ADON). Kernel inoculum was dispersed at a rate of 8 g per row on biweekly intervals, starting at Zadoks stage 31. The application of the inoculum was followed by irrigation three times a week using Cadman Irrigations Travellers with Briggs booms. Visual observations were taken at 18–21 days post inoculation for infected heads (disease incidence; DI) and spikelets (disease severity; DS) using a 0 to 10 scale, which were used to calculate FHB visual rating index (VRI: DI × DS) (Gilbert and Morgan, 2000). Wheat plots were manually harvested and threshed using a stationary combine, then seed was manually cleaned to prevent the loss of Fusarium-damaged kernels.

In Ottawa, a *Fg* inoculum was prepared with 1:1 corn and barley kernels inoculated with three *Fg* isolates: DAOMC178148 (15-ADON), DAOMC212678 (15-ADON), and DAOMC232369 (3-ADON) sourced from the Canadian Collection of Fungal Cultures at the Ottawa Research and Development Centre (ORDC). Isolates were chosen from those collected locally with high deoxynivalenol (DON) producing capacity. Inoculum was prepared as described in Xue et al. (2006). Inoculation with 12 g per line of fresh inoculum was performed twice, first application occurring when the earliest lines started stem elongation, before flag leaf emergence (Zadoks stage 31–36), and again 2 weeks later. Plots were irrigated daily applying approximately 1.5 cm of rain equivalent with wedge drive impact sprinklers. Flowering date (50% flowering) were recorded for each plot, and visual observations were made 21 days after flowering for each plot. Rating scales, harvest, and sample threshing were performed as in Morden (see description above).

For DON analysis, to make a whole-grain flour, one 25 g aliquot from the two replications of each cultivar was ground with a Perten Laboratory mill 3,310 to pass through a 0.4-mm screen. A single 1 g ground sub-sample was taken and extracted with 5 mL of methanol:water (1:9, vol:vol) in 10-mL plastic tubes, which were then subjected to end-over-end mixing for 1 hour, centrifuged for 5 min at 2000 rpm. DON analysis was conducted on the filtrate using the in-house enzyme-linked immunosorbent assay (ELISA) as described by Sinha et al. (1995). The accuracy of the ELISA procedures has been reported to be comparable to that of the gas chromatography method (Sinha and Savard, 1996). The limit of quantitation was 0.1 mg kg^−1^.

### 2.7 Genotyping

Genomic DNA bulks (10 plants/cultivar) were extracted from greenhouse-grown young leaf tissue with the Macherey-Nagel NucleoSpin 96 Plant II kit (Macherey-Nagel GmbH and Co. KG, Düren, Germany). dsDNA concentrations were determined using the fluorescence-based Quant-IT dsDNA Broad Range Assay kit (ThermoFisher Scientific, cat #Q33130) on the BMG FLUOstar Omega microplate reader with Omega MARS data analysis software (BMG Labtech GmbH, Ortenburg, Germany). The DNA bulks were diluted with sterile, distilled water to working concentrations of 10 ng/μL.

KASP markers were from the University of Bristol wheat MAS set (http://www.cerealsdb.uk.net/cerealgenomics/CerealsDB/kasp_download.php?URL/MAS_data_May_2013.xls) and the references listed in Table 1. All primers were commercially prepared (Invitrogen) from sequences obtained from the literature.

**TABLE 1:** Molecular markers analysed in the Brazilian material to predict the presence of specific loci/genes (Röder et al., 1998; Dreisigacker et al., 2016).

Trait	Locus/Gene	Marker	Marker type	Reference
Combined resistance	*Lr16/Sr23*	*kwm847*	KASP	Kassa et al. (2017)
	*Lr34/Yr18/Pm38/Sr57/Ltn1*	*cssfr5*	agarose gel-based	Lagudah et al. (2009)
		wMAS000003	KASP	http://maswheat.ucdavis.edu/, http://www.cerealsdb.uk.net/
	*Lr37/Yr17/Sr38*	2AS_VPM_CAPS	STS	Helguera et al. (2003)
	*Lr67/Yr46/Sr55/Pm46/Ltn3*	*csSNP856*	KASP	Forrest et al. (2014)
	*Sr2/Yr30*	wMAS000005	KASP	http://maswheat.ucdavis.edu/, http://www.cerealsdb.uk.net/
		*csSr2*-CAPS	CAPS, agarose gel-based	Mago et al. (2011b)
	*SrCad/Sr42/Bt10*	*kwm907*	KASP	Kassa et al. (2016)
*Wheat–rye translocation*	*1RS:1BL- Lr26, Sr31, Yr9, Pm8*	wMAS0000011	KASP	http://maswheat.ucdavis.edu/, http://www.cerealsdb.uk.net/
Fusarium head blight	*Fhb1 (3BS)* (Sumai-3)	*TaHRC*-KASP	KASP	Su et al. (2018)
		UMN10	STS	Liu et al. (2008)
		*gwm493*	SSR	McCartney et al. (2004), Bernardo et al. (2011)
		*gwm533*	SSR	
		wMAS000008	KASP	http://maswheat.ucdavis.edu/, http://www.cerealsdb.uk.net/
		wMAS000009	KASP	
	*Fhb2 (6BS)* (Sumai-3)	*GBS0158*_6BS	KASP	Cai et al. (2016)
		*wmc397*	SSR	McCartney *et al.* (2004), Cuthbert et al. (2007)
		*wmc398*	SSR	
		*gwm508*	SSR	
		*gwm133*	SSR	
		*gwm644*	SSR	
	Qfhs.ifa-5AS (Sumai-3)	*IWA7777*	KASP	Pandurangan et al. (2021)
	Qfhi.nau-5AS (Wangshuibai, Sumai 3, Frontana)	*gwm415*	SSR	Steiner et al. (2004), (2019), Xue et al. (2011)
		*gwm304*	SSR	
		*gwm293*	SSR	
		*wmc96*	SSR	
	*QTL Fusarium 5A* (Haiyanzhong)	*GBS 1852*_5A	KASP	Cai et al. (2016)
	Qfhs.ifa-5Ac (Sumai-3)	*wmc705*	SSR	Buerstmayr *et al.* (2003), McCartney *et al.* (2004), Steiner *et al.* (2019), Pandurangan *et al.* (2021)
		*barc180*	SSR	
	*QTL_3AL* (Frontana)	*gwm1110*	SSR	Steiner et al. (2004), Marion Röder, pers. comm.
		*gwm720*	SSR	
		*gwm1121*	SSR	
Plant height	*Rht8*	*gwm261*	SSR	Ellis *et al.* (2007)
	*Rht-B1*	wMAS000001	KASP	http://maswheat.ucdavis.edu/, http://www.cerealsdb.uk.net/, Ellis et al. (2002)
	*Rht-D1*	wMAS000002	KASP	http://maswheat.ucdavis.edu/, http://www.cerealsdb.uk.net/, Ellis et al. (2002)

KASP reactions were assembled in either 96 (Bio-Rad, cat # HSP9655) or 384 well (4Titude, cat # 4ti-0387) PCR plates, following LGC Biosearch Technologies’ recommended protocol (https://biosearch-cdn.azureedge.net/assetsv6/KASP-genotyping-chemistry-User-guide.pdf). KASP assay mix was prepared according to Smith and Maughan (Smith and Maughan, 2015). For the 96 well format, each PCR reaction contained 5 µL genomic DNA (10 ng/μL), 5 µL KASP 2x MasterMix (standard ROX, BioSearch Laboratories cat # KBS-1050-102) and 0.14 µL KASP assay mix containing two allele specific primers and one common primer (Invitrogen). For the 384 well format, each well contained 2.5 µL genomic DNA (10 ng/μL), 2.5 µL KASP MasterMix and 0.07 µL KASP assay mix. PCR amplifications were carried out in either a Bio-Rad C1000 Touch (Bio-Rad Laboratories (Canada) Ltd., Mississauga, ON), Eppendorf Master Cycler Gradient (Eppendorf Canada, Mississauga, ON) or Veriti 384 (Applied Biosystems, Foster City, CA, United States) thermal cyclers, following the recommended touchdown thermal cycling conditions. Fluorescence readings were performed in a multimode microplate reader: Spark 10M (Tecan, Männedorf, Switzerland) or FLUOstar Omega (BMG Labtech GmbH, Ortenburg, Germany). Data were imported into KlusterCaller genotyping software (LGC Biosearch Technologies, Hoddeston, Herts, United Kingdom) for analysis.

For SSR and STS markers resolved by capillary electrophoresis, the total PCR reaction volume was 20 µL. For SSR markers, each PCR reaction contained: 50 ng genomic DNA, 180 nM dye-labelled M13 primer (5′- CAC GAC GTT GTA AAA CGA C, labelled at the 5′ end with one of 6-FAM, PET, VIC, or NED), 20 nM M13 sequence-labelled forward primer, 200 nM unlabelled reverse primer, 1X *Taq* buffer containing (NH_4_)_2_SO_4_ (ThermoScientific, cat #B33), 1.5 mM MgCl_2_, 200 µM each dNTP and 1U *Taq* DNA polymerase (DreamTaq, ThermoScientific).

For the STS marker UMN10 (Liu et al., 2008) at *Fhb1*, the PCR conditions described by Liu and Anderson (2003) were adapted for capillary electrophoresis. Each PCR reaction contained: 50 ng genomic DNA, 50 mM dye-labelled (6-FAM or PET) M13 primer, 50 nM M13-labelled forward primer, 100 nM reverse primer, 1X *Taq* buffer containing (NH_4_)_2_SO_4,_ 1.5 mM MgCl_2_, 200 µM each dNTP and 1U *Taq* DNA polymerase.

Marker 2AS_VPM_CAPS, diagnostic for the 2NS segment from *Triticum ventricosum* (Helguera et al., 2003), was also adapted for capillary electrophoresis. Each PCR reaction contained: 50 ng genomic DNA, 50 nM 6-FAM-labelled M13 primer, 50 nM M13-labelled URIC forward primer, 100 mM unlabelled reverse primer LN2, 1X *Taq* buffer containing (NH_4_)_2_SO_4_, 1.5 mM MgCl_2_, 200 µM each dNTP and 1U *Taq* DNA polymerase. PCR products were not digested with a restriction enzyme.

PCR cycling occurred in one of the above-mentioned thermal cyclers, with the marker-specific conditions detailed in the literature (Table 1). Amplification products were resolved by capillary electrophoresis using the ABI 3130xl or 3,500 Genetic Analyzers, with GeneScan 600 LIZ v. 2.0 (Applied Biosystems, Foster City, CA, United States) as the sizing standard. Alternatively, the PCR products were sent to “Centre d’expertise et de services Génome Québec” to be resolved on an Applied Biosystems 3730XL DNA analyzer. SSR and STS marker data were analyzed using GeneMapper 5 software (Applied Biosystems).

PCR and agarose gel protocols for *cssfr5* (Lagudah et al., 2009) and csSr2_CAPS (Mago et al., 2011a) markers were as described in the literature.

### 2.8 Statistics

Agrobase Generation II (Agronomix Software Inc., Winnipeg, MB) was used to calculate the mean, least significant difference (LSD) and coefficient of variation (CV) of traits from agronomic field trials and Fusarium nurseries. RStudio (RStudio Team, 2015) and ggplot2 package (Wickham, 2016) were the main tools used for statistical calculation and plotting the results. Grain yield by grain protein was plotted using the libraries ggplot2 (Wickham, 2016), dplyr (Revelle, 2022), hrbrthemes (Rudis et al., 2020), viridis (Garnier et al., 2021), ggrepel (Slowikowski et al., 2021) and ggpmisc (Aphalo, 2022). Those libraries were also used to calculate and plot the bubble charts of leaf diseases and traits-related with FHB. Pearson’s correlation coefficients were calculated for traits related with FHB using psych (Revelle, 2022), and plotted using corrplot (Wei and Simko, 2021) packages in R. Boxplots representing severity for each cultivar to leaf rust, stripe rust, stem rust and powdery mildew were computed and plotted with ggplot2 (Wickham, 2016) and stat_summary. Those were also used to calculate and to prepare the boxplot charts of FHB traits classified by collection. Ggarrange function in ggpubr package (Kassambara, 2020) was used to combine ggplot2 charts in a same figure. The percent stacked bar chart was designed with ggplot2 (Wickham, 2016).

## 3 Results

### 3.1 Germplasm and agronomic performance

**FIGURE 1:**
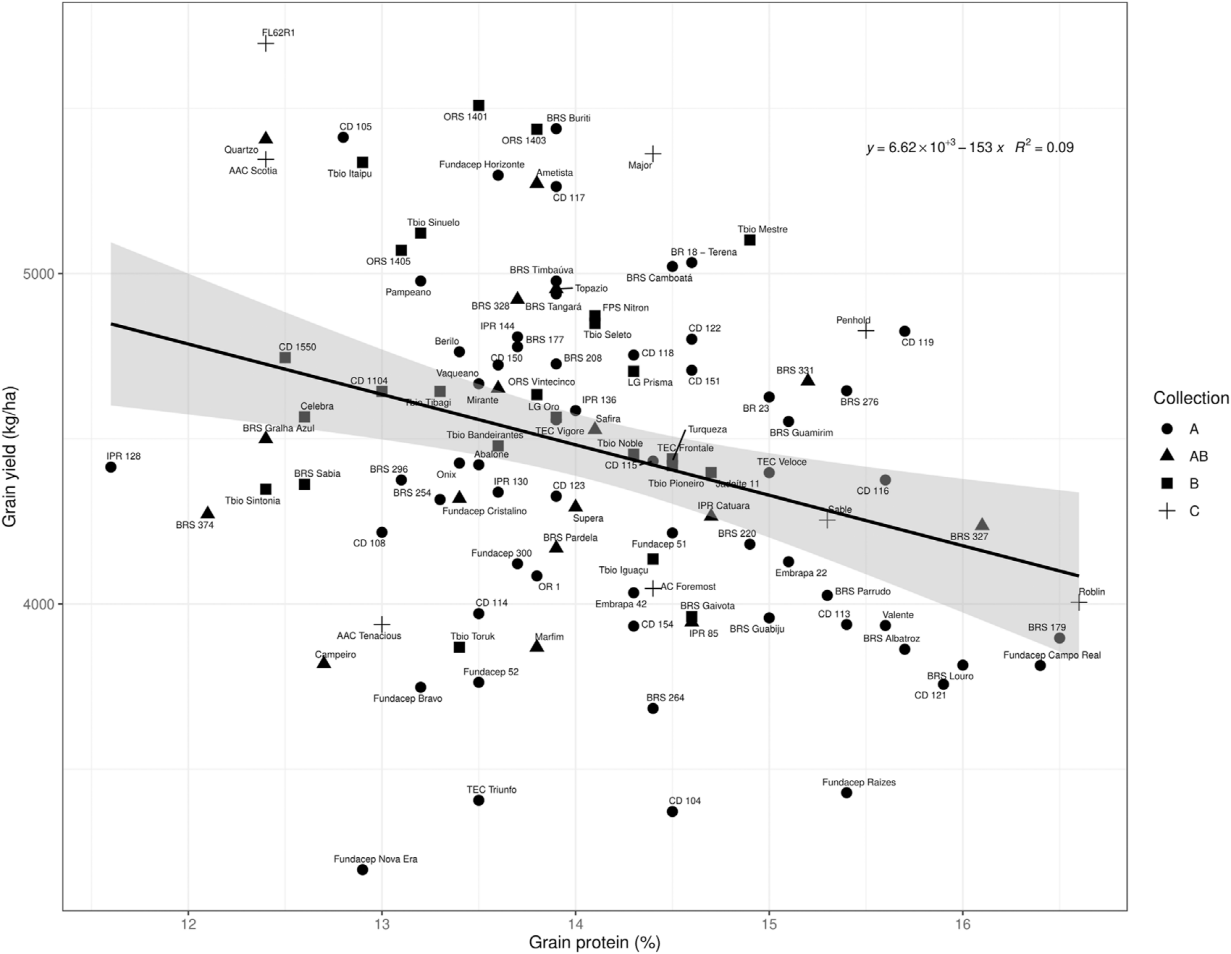
Grain yield by grain protein plot of the Brazilian collections and Canadian checks evaluated at Saint-Mathieu-de-Beloeil in 2018. The material was divided in collections: “A”—cultivars registered in Brazil from 1986 to 2012, but not recommended for cultivation in Brazil after 2015; “B”: cultivars registered from 1999 to 2016 and still in recommendation to be cultivated in 2015; “AB”—cultivars present in both collections, still in recommendation after 2015. “C” represents Canadian checks. The linear regression line is in black and the confidence interval (0.95) is displayed in gray.

There was a considerable parental diversity in the pedigrees of the collection cultivars which suggests there is high degree of genetic variability present in the material (Supplementary Table S1). Cultivars varied widely with respect to agronomic performance as indicated by the wide range of grain yield, thousand kernel weight, test weight, heading date, maturity, plant height and protein content (Table 2). Some of the Brazilian cultivars performed well in eastern Canada. In Ottawa 2017, the highest yielding cultivar was Celebra from the collection, and 30 other Brazilian cultivars were superior or statistically equal to the highest yielding Canadian check, AAC Scotia. In Beloeil 2018, the Canadian line FL62R1 produced the highest grain yield; however, 10 Brazilian cultivars had statistically similar grain yield (Figure 1). Over both years, the Brazilian collection averaged higher mean thousand kernel weight and mean test weight, later mean maturity, shorter mean plant height compared to the mean of the Canadian checks.

**TABLE 2:** Mean of trial, maximum mean among Brazilian cultivars and among Canadian checks, range, least significant difference (LSD) and coefficient of variation (CV) of agronomical traits in two field trials in east Canada in 2017 (Ottawa, ON) and 2018 (St-Mathieu-de-Beloeil, QC).

	Yield (kg/ha)		Thousand kernel weight (g)		Test weight (kg/hL)		Heading (days)		Maturity (days)	Height (cm)		Protein (%)	
	2017	2018	2017	2018	2017	2018	2017	2018	2018	2017	2018	2017	2018
Trial mean	2,223	4,471	24.6	34.9	70.8	72.5	196.1	54.0	81.2	81.6	69.1	12.5	14.0
Max mean - BR cultivars	3,310	5,508	36.5	46.4	76.9	75.5	74.0	60.0	89.5	105.0	82.0	15.0	16.5
Max mean - CA checks	2,729	5,696	31.6	42.7	73.7	74.1	69.0	58.0	84.5	111.0	96.0	14.8	16.6
Range	2,634	2,501	21.6	17.1	16.3	4.7	17.5	13.0	17.5	49.0	41.0	4.4	5.0
LSD	244.7	645.0	4.6	2.0	3.4	3.0	1.8	2.3	4.5	10.3	5.9	1.1	1.2
CV	4.6	6.1	7.9	2.4	2.0	1.7	0.4	1.8	2.4	5.3	3.6	3.8	3.7

Grain yield and grain protein content were significantly negatively correlated in the 2018 trial (Pearson correlation: −0.88, *p* = 0.02), but not in the 2017 trial (Pearson correlation: −0.63, *p* = 0.18). Cultivars from Brazilian collection “B” generally had higher grain yield and intermediate protein content compared to collection “A” (Figure 1).

### 3.2 Rusts and powdery mildew

**FIGURE 2:**
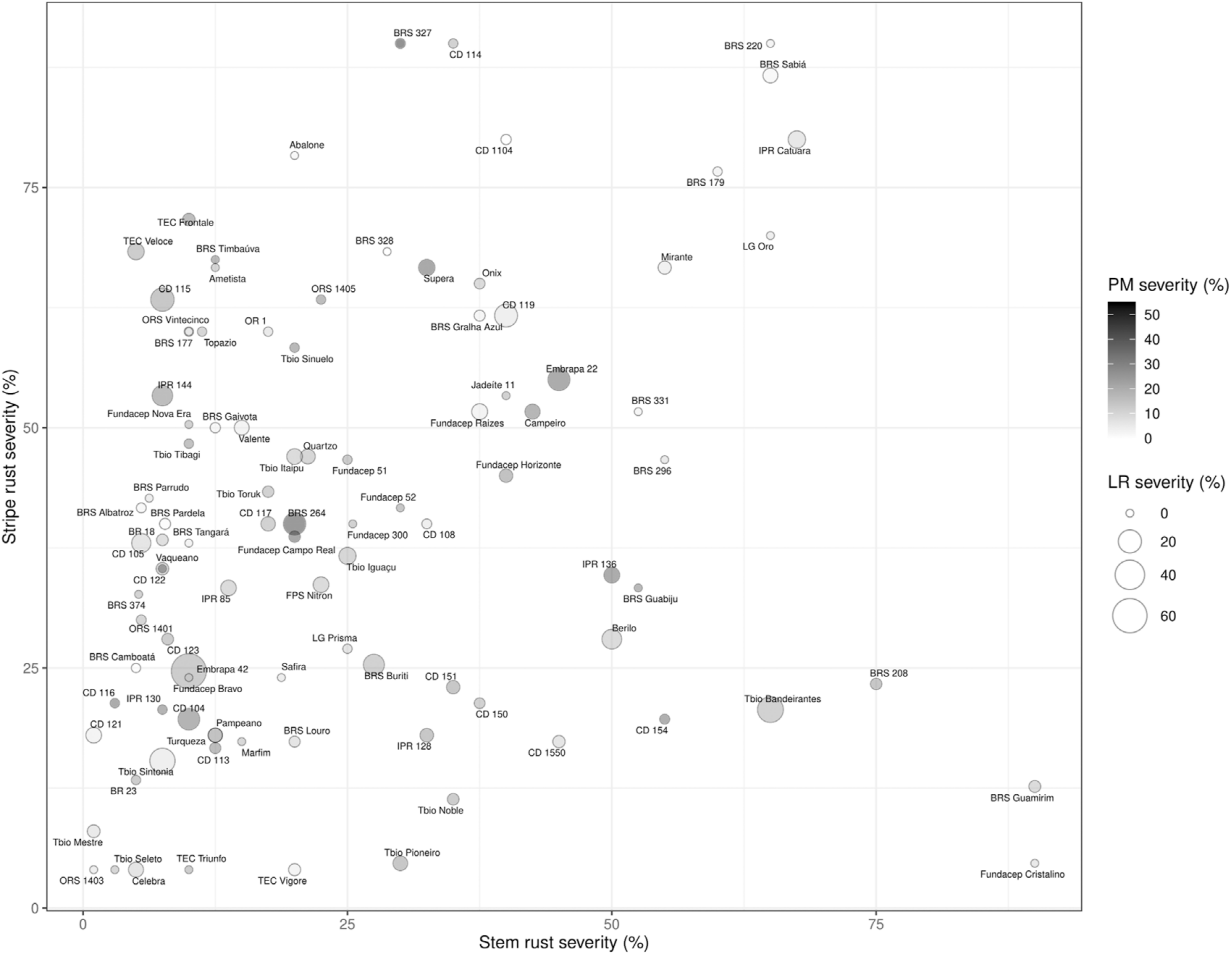
Chart representing the average reaction of the Brazilian cultivars to leaf rust (bubble size), stripe rust (y-axis), stem rust (x-axis) and powdery mildew (color intensity) in field nurseries in Canada.

Good disease pressure was observed in the leaf rust nurseries which was demonstrated by the high mean severity of the susceptible check, AC Foremost (76.7% and 60% in 2017 and 2018, respectively). Brazilian cultivars showed good resistance to Canadian leaf rust isolates (Figure 2; Table 3) with over 80% of Brazilian cultivars having a severity of 10% or less. At the seedling stage, 43 Brazilian cultivars were resistant (scored below “3”) to the six Canadian races (Table 3). Only five (BR 18, BRS Pardela, CD 104, Pampeano and TEC Vigore) were susceptible at seedling stage to all six races; however, these cultivars appeared to possess adult leaf rust resistance. According to the molecular markers results, none of these five cultivars possess *Lr34*, *Lr37*, *Lr16*, *Lr26,* or *Lr67*; except TEC Vigore that has *Lr16*. Adult plant resistance (APR), not based on *Lr34* or *Lr67,* was observed in the Brazilian cultivars Abalone, ORS 1401, ORS 1403 and Safira (Table 3).

**TABLE 3:** Characterization of the Brazilian collections to leaf rust (*Puccinia triticina*). Reaction at seedling stage of Brazilian wheat cultivars to Canadian leaf rust isolates, comparison of resistance reaction between Canada and Brazil (Dotto et al., 2005; Informações técnicas para trigo e triticale - Safra, 2012, 2011; Cunha and Caierão, 2014; Cunha et al., 2016; Silva et al., 2017; Franco and Evangelista, 2018; Informações técnicas para trigo e triticale - Safra, 2019, 2018; Informações técnicas para trigo e triticale - Safra, 2020, 2020; Informações técnicas para trigo e triticale - Safra, 2022, 2022). Prediction of the resistance genes in the collection by genotypage of genes/loci related with leaf rust resistance. APR: adult plant resistance determined phenotypically by comparing seedling and adult plant resistance (no indication of APR presence in the table does not mean necessarily absence of APR); pos: positive allele, presence of the resistant allele; neg: negative, presence of the susceptible allele; hetero: heterozygosity in the locus; NSD: not sufficient data.

Cultivar	*Canadian P. triticina* isolates—seedling stage						Leaf rust severity (%) in CA field^a^	Brazil field leaf rust classification^b^		Molecular markers				
	MBDS	MBRJ	MGBJ	11-TDBG	TJBJ	06-TDBG				*Lr16*	*Lr34*	*Lr37*	*Lr67*	*Lr26 (1RS:1BL)*
Abalone					3+		0.0	MRMS	APR	neg	neg	neg	neg	neg
Ametista				;1/4	4	;1-	0.0	MR		neg	neg	neg	neg	neg
Berilo	4	4	4	;1/x	3+	x	11.8	MR		neg	neg	neg	neg	neg
BR 18 - Terena	4	4	3+	3+	3+	4	1.2	MS		neg	neg	neg	neg	neg
BR 23	;1-		;1		2+		0.2	APR	APR	neg	pos	neg	neg	pos
BRS 177				;1+	2+	;1 =	0.2	SMS		neg	neg	neg	neg	hetero
BRS 179	3		;1-	;1+	2-	;1 =	0.2	S		neg	neg	neg	neg	pos
BRS 208	0			0	0	0	1.0	R		neg	pos	neg	neg	neg
BRS 220				0		0	0.0	MS		neg	neg	neg	neg	pos
BRS 296	4	;1	x	;1 2-	3+	3	0.0	-	APR	neg	pos	neg	neg	neg
BRS 327	x		;1-	;1-	;1-	;1-	0.3	S		neg	neg	neg	neg	pos
BRS 328				;1+	x	2+	0.0	MRR		neg	neg	neg	neg	neg
BRS 331		;1 =		;1 =	2-	;1 =	0.0	MSMR		neg	neg	neg	neg	pos
BRS 374	4	4	4	;1 =	2-	x	0.0	S		neg	neg	neg	neg	neg
BRS Albatroz	3+	3+	2 3+	3+	3+	3+	0.3	MS		neg	neg	neg	neg	pos
BRS Buriti				;1	1+	2+	14.5	-		neg	neg	neg	neg	neg
BRS Camboatá				x	3+	4	0.2	-		neg	pos	NSD	neg	neg
BRS Gaivota	;1-	;1 =	;1 =	;1 =			0.5	MS		neg	neg	neg	neg	pos
BRS Gralha Azul	2 + 3	4	2+	;1 =	2+	2+	0.8	MR		neg	neg	neg	neg	neg
BRS Guabiju	2	3+	2 3	2+	3+	4	0.0	MRMS		neg	neg	neg	neg	neg
BRS Guamirim		0			;/3+	;/2	1.3	MRMS		neg	pos	neg	neg	neg
BRS Louro				2+	3+	1-	0.8	MS		neg	neg	neg	neg	pos
BRS Pardela	3	3+	3+	3+	3+	4	0.8	MR		neg	neg	neg	neg	neg
BRS Parrudo		;1 =		;1	4	;1-	0.0	MRMS		neg	neg	neg	neg	pos
BRS Sabiá	;1 =		X	;1 =	;1 =		4.2	MS		neg	pos	neg	neg	neg
BRS Tangará					2	4	0.0	R		neg	pos	neg	neg	neg
BRS Timbaúva				;1-	3+	;1 =	0.0	-		neg	neg	neg	neg	neg
Campeiro	0	0	0	;1 2-	3+	4	4.3	MRMS		neg	neg	neg	neg	neg
CD 104	4	3	3+	4	3+	3+	15.8	S		neg	neg	neg	neg	neg
CD 105	4	2 3	3+	3+	3+	3+	10.8	MS		neg	neg	neg	neg	neg
CD 108				;1 =	3+	;1-/3+	0.3	MR		neg	neg	neg	neg	neg
CD 1104	0				2	0	0.5	MS		neg	neg	neg	neg	neg
CD 113	;1 =	0		;1 =	3+	;1-/3+	0.8	-		neg	neg	neg	neg	neg
CD 114	0	0	0	;1-	3+	;1-	0.2	MR		neg	neg	neg	neg	pos
CD 115		0	0		0	0	20.3	MR		neg	neg	neg	neg	neg
CD 116	2-	;1-	;1-	;1-	3+	;1-	0.2	MR		pos	neg	pos	neg	neg
CD 117	3	;1	;1+	2-	2+	;1 =	3.5	MS		neg	neg	neg	neg	neg
CD 119				4	3	3+	19.2	MS		neg	neg	neg	neg	neg
CD 121	0		0	3+	4	4	5.2	MR		NSD	neg	hetero	neg	neg
CD 122				1+	2	2	1.8	MR		NSD	neg	hetero	neg	neg
CD 123	;/3		x	;1/x	x	x	1.2	MR		neg	neg	neg	neg	neg
CD 150	4	;1/x	1 2	x	4	x	0.7	MR		neg	neg	neg	neg	neg
CD 151	;1-/3		;1+	3+	4	3+	2.5	MS		neg	neg	neg	neg	neg
CD 154	2+	1+	;1+	;1	2+	;1-	0.3	MS		pos	neg	neg	neg	neg
CD 1550	;1 =	;1 =			;1 =		1.7	MR		neg	neg	neg	neg	neg
Celebra	2-	;1-	;1 =		23		4.3	MS		neg	neg	hetero	neg	neg
FPS Nitron	0	0	0	;1 =	;1 =		5.2	S		neg	hetero	neg	neg	neg
Fundacep 300	;1		;1-		2-	0	0.0	S		neg	neg	neg	neg	pos
Fundacep 51	;1		;1-		2-		0.2	S		neg	neg	neg	neg	pos
Fundacep 52	;1		;1-	;1-	2-	;1 =	0.0	S		neg	neg	neg	neg	pos
Fundacep Bravo			;1 =	;1-	2-	;1 =	0.0	MR		neg	neg	neg	neg	hetero
Fundacep Campo Real			0	0		0	1.0	S		neg	neg	neg	neg	neg
Fundacep Cristalino	3	2	;1 =	2	3+	2-	0.0	MR		pos	neg	neg	neg	neg
Fundacep Horizonte		;1+	0		3+	3	2.5	R		neg	neg	neg	neg	neg
Fundacep Nova Era	;1-		;1 =	;1 =	2-		0.0	S		neg	neg	neg	neg	pos
Fundacep Raizes	2 3	2 3	2 3	2 3	3+	4	5.5	MR		neg	neg	neg	neg	neg
IPR 128	2/3	1+	1 2	;1 2	4	;1 =	2.7	MS		neg	neg	neg	neg	neg
IPR 130	3+	;1-		;1-	x		0.2	MS		neg	neg	neg	neg	neg
IPR 136	0		0	;1-	3+	;1 =	5.2	MS		NSD	neg	neg	neg	neg
IPR 144	2+	1++	3	1++	3+	3+	13.3	MR		hetero	neg	neg	neg	neg
IPR 85	3	;1-	;1-	;1-	2+	2-	5.0	MR		neg	neg	neg	neg	neg
IPR Catuara			0	x	3+	3+	7.7	S		neg	neg	neg	neg	neg
Jadeíte 11	0	0	0	1-	2-	0	0.0	MRR		neg	neg	neg	neg	neg
LG Oro		0	;1 =	0	12		0.0	MR		neg	neg	neg	neg	neg
LG Prisma	0	0	0		0	0	0.2	R		NSD	pos	neg	neg	neg
Marfim	4	4	3+	;1/3+	3+	x	0.0	MR		neg	neg	neg	neg	neg
Mirante	2++	;1+	1 + 2	;1-	3+	;1 =	2.3	S		neg	neg	neg	neg	neg
Onix	2	;1		;1+	3+	;1 =	0.7	S		neg	neg	neg	neg	neg
OR 1	;1 =			;1 2-	3+	;1 =	0.2	-		neg	neg	neg	neg	neg
ORS 1401	0	0	0	;1 =	;1 =	1-	0.3	RMR	APR	neg	neg	pos	neg	neg
ORS 1403	0			;1 =	;1 =	;1 =	0.0	RMR	APR	neg	neg	pos	neg	neg
ORS 1405	0				;1 =		0.2	SMS		neg	neg	NSD	neg	neg
ORS Vintecinco		;1 =	;1 =	;1 =	;1 =		0.0	RMR		neg	neg	neg	neg	neg
Pampeano	4	4	3	4	3+	4	3.7	-		neg	neg	neg	neg	neg
Quartzo			0	3+	3+	2-/3+	4.2	MS		neg	neg	neg	neg	neg
Safira				;1	3+	;1 =	0.0	MS	APR	neg	neg	neg	neg	neg
Supera	3	2	2+3-	;1+	3+	;1-	6.0	MS		neg	neg	neg	neg	neg
Tbio Bandeirantes	3	;1 =	3+	3	3+	3-	27.5	MS		neg	neg	NSD	neg	neg
Tbio Iguaçu	0	0/2	0	2-	1 =	2-	7.2	MS		neg	neg	NSD	neg	neg
Tbio Itaipu	0	0	0	;1 =	;1 =	;1 =	5.2	MS		neg	neg	NSD	neg	neg
Tbio Mestre		0		;1 =	2-	;1 =	2.0	R MR		neg	neg	NSD	neg	neg
Tbio Noble	;1 =	23	;1 =	;1 =	12	0	1.2	-		neg	neg	neg	neg	neg
Tbio Pioneiro	;1 =	;1 = /3	;1 =	0	;1 =		3.8	MR		neg	neg	hetero	neg	neg
Tbio Seleto	0	0	0		;1 =		0.0	MS		neg	pos	neg	neg	neg
Tbio Sintonia	12	;1 =	;1-		2-		25.8	MS		neg	neg	NSD	neg	neg
Tbio Sinuelo	0	0			;1 =		0.2	MR		neg	neg	neg	neg	neg
Tbio Tibagi	;1 =	2-	;1 =	;1 =	;1 =	;1 =	0.2	S		neg	neg	neg	neg	neg
Tbio Toruk	0	2-	;1-	2-	2-	1-	1.0	MR		neg	neg	neg	neg	neg
TEC Veloce				4	4	4	6.2	S		neg	neg	neg	neg	neg
TEC Vigore	3	4	4	4	4	4	1.5	MRR		pos	neg	neg	neg	neg
Topazio				;1-	2+	;1−	0.2	-		neg	neg	neg	neg	neg
Turqueza				;1 = /4	4	4	3.2	MRMS		neg	neg	neg	neg	neg
Valente	x/3	2	x/3	;1	4	;1−	4.2	MS		neg	hetero	neg	neg	neg
Vaqueano				;1 =	4		0.0	MR		neg	neg	neg	neg	neg
AAC Scotia	4	4	4	3+	4	4	15.8	-		neg	neg	neg	neg	neg
AAC Tenacious	3	3+	4	x	4	x	0.3	-		neg	NSD	NSD	neg	NSD
AC Foremost	3	3+	3+	3+	2+	4	68.3	-		neg	neg	NSD	neg	neg
FL62R1	0	0	0	2+	2+	2+	0.2	-		neg	pos	neg	neg	neg
Penhold	2 + 3	;1 =	0	2-	1-	;1 =	2.7	-		neg	NSD	NSD	neg	NSD
Roblin	3	4	3+	4	3+	4		-		hetero	NSD	NSD	neg	NSD
Sable	3+	3+	3+	;1-	4	x	3.3	-		neg	NSD	NSD	neg	NSD

^a^ Morden 2017–2018 Field Canada.

^b^ Informações Técnicas para Trigo e Triticale, disease reaction provided by cultivar’s sponsor.

Markers associated with wheat rust resistance or multiple disease resistance loci such as *Lr34/Yr18/Pm38/Sr57/Ltn1*, *Lr67/Yr46/Sr55/Pm46/Ltn3*, *Lr37/Yr17/Sr38*/blast, *Lr16/Sr23*, *Sr2/Yr20/Lr27*/PM/pseudo-black chaff, and *SrCad*/*Bt10* were evaluated (Tables 3, 4 and 5). The frequency of the resistant alleles (Figure 6) to those genes were below 25% in the Brazilian collections, and *Lr67* and *SrCad* were completely absent. *Lr34* was present in only 10 cultivars, and *Lr16* was identified in 5 cultivars. The presence of the *T. ventricosum* 2NS translocation, which confers blast resistance besides rust resistance, was present in CD 116, ORS 1401 and ORS 1403, while CD 121, CD 122, Celebra and Tbio Pioneiro were showed to be heterogeneous for the translocation.

**TABLE 4:** Characterization of the Brazilian collections to stem rust (*Puccinia graminis*). Reaction at seedling stage of Brazilian wheat cultivars to Canadian stem rust isolates and TTKSK, and field data in Canada. Prediction of the resistance genes in the collection by genotypage of genes/loci related with stem rust resistance. Pos: positive allele, presence of the resistant allele; neg: negative, presence of the susceptible allele; H: heterozygosity in the locus; NSD: not sufficient data; R: resistance; MR: moderate resistance; I: intermediate; MS: moderate susceptibility; S: susceptibility.

Cultivar	Seedling test - Canadian *P. graminis* isolates and TTKSK										Field Canada^a^		Molecular markers						
	QCC	TPM	RHT	TMR	RKQ	QTH	RTH	MCC	QFC	TTKSK	Severity	Infection type	*Sr23 (Lr16)*	*Sr57 (Lr34)*	*Sr38 (2NS)*	*Sr55 (Lr67)*	*SrCad/Bt10*	*Sr2/Yr30*	*Sr31* (1RS:1BL)
Abalone	-	-	-	-	-	-	-	-	-	-	20	MR-I	neg	neg	neg	neg	neg	neg	neg
Ametista	0	1-	0	1–1	0	1-	1-	0	;1-	;1-/33-	12.5	MR	neg	neg	neg	neg	neg	neg	neg
Berilo	-	-	-	-	-	-	-	-	-	-	50	MS	neg	neg	neg	neg	neg	neg	neg
BR 18 - Terena	-	-	-	-	-	-	-	-	-	-	7.5	R-I	neg	neg	neg	neg	neg	neg	neg
BR 23	-	-	-	-	-	-	-	-	-	-	5	R-MS	neg	pos	neg	neg	neg	neg	pos
BRS 177	-	-	-	-	-	-	-	-	-	-	10	MR	neg	neg	neg	neg	neg	neg	H
BRS 179	-	-	-	-	-	-	-	-	-	-	60	I-MS	neg	neg	neg	neg	neg	neg	pos
BRS 208	-	-	-	-	-	-	-	-	-	-	75	I-S	neg	pos	neg	neg	neg	neg	neg
BRS 220	-	-	-	-	-	-	-	-	-	-	65	MS-S	neg	neg	neg	neg	neg	neg	pos
BRS 296	-	-	-	-	-	-	-	-	-	-	55	MR-S	neg	pos	neg	neg	neg	neg	neg
BRS 327	0	1-	0	0	0	0	0	0	0	0	30	I-MS	neg	neg	neg	neg	neg	neg	pos
BRS 328	;1-	1-	0	1-	0	1-	1-	1–1	1–1	1-	28.8	MR-MS	neg	neg	neg	neg	neg	neg	neg
BRS 331	1-	0	0	1-	0	1-	1-	1-	1-	1-	52.5	I-S	neg	neg	neg	neg	neg	neg	pos
BRS 374	;1-	0	0	1-	0	0	0	0	1-	;1-	5.25	MR-R	neg	neg	neg	neg	neg	neg	neg
BRS Albatroz	-	-	-	-	-	-	-	-	-	-	5.5	MR-R	neg	neg	neg	neg	neg	neg	pos
BRS Buriti	-	-	-	-	-	-	-	-	-	-	27.5	MR-I	neg	neg	neg	neg	neg	neg	neg
BRS Camboatá	-	-	-	-	-	-	-	-	-	-	5	R-MR	neg	pos	NSD	neg	neg	neg	neg
BRS Gaivota	;1-	1-	0	1-	0	1-	1-	0	;1-	1-	12.5	MR-I	neg	neg	neg	neg	neg	neg	pos
BRS Gralha Azul	1–1	1-	1-	1-	0	1	1	1-	1	1-	37.5	I-MR	neg	neg	neg	neg	neg	neg	neg
BRS Guabiju	-	-	-	-	-	-	-	-	-	-	52.5	I-S	neg	neg	neg	neg	neg	neg	neg
BRS Guamirim	-	-	-	-	-	-	-	-	-	-	90	R-S	neg	pos	neg	neg	neg	neg	neg
BRS Louro	-	-	-	-	-	-	-	-	-	-	20	I-I	neg	neg	neg	neg	neg	neg	pos
BRS Pardela	;1-	1-	;1-	1-	;1-	1-	1–1	0	;1-	34	7.75	R-MR	neg	neg	neg	neg	neg	neg	neg
BRS Parrudo	1-	1-	1-	1-	0	1-	1-	1-	1–1	;1-	6.25	R-I	neg	neg	neg	neg	neg	neg	pos
BRS Sabiá	1–1	1-	;1-	0	1-	1-	1-	1-	11+	1-	65	MS-S	neg	pos	neg	neg	neg	neg	neg
BRS Tangará	-	-	-	-	-	-	-	-	-	-	10	MR	neg	pos	neg	neg	neg	neg	neg
BRS Timbaúva	-	-	-	-	-	-	-	-	-	-	12.5	I-MR	neg	neg	neg	neg	neg	neg	neg
Campeiro	0	1	1-	1-	1-	1-	1-	1–1	1-	3+-	42.5	MS-I	neg	neg	neg	neg	neg	neg	neg
CD 104	-	-	-	-	-	-	-	-	-	-	10	I-MS	neg	neg	neg	neg	neg	neg	neg
CD 105	-	-	-	-	-	-	-	-	-	-	5.5	MR-R	neg	neg	neg	neg	neg	neg	neg
CD 108	-	-	-	-	-	-	-	-	-	-	32.5	I	neg	neg	neg	neg	neg	neg	neg
CD 1104		1	0	1-	1-	1-	0	0	1-		40	MS-S	neg	neg	neg	neg	neg	neg	neg
CD 113	-	-	-	-	-	-	-	-	-	-	12.5	MR-I	neg	neg	neg	neg	NSD	neg	neg
CD 114	-	-	-	-	-	-	-	-	-	-	35	MS-I	neg	neg	neg	neg	neg	neg	pos
CD 115	-	-	-	-	-	-	-	-	-	-	7.5	I	neg	neg	neg	neg	neg	neg	neg
CD 116	-	-	-	-	-	-	-	-	-	-	3	R-MR	pos	neg	pos	neg	neg	neg	neg
CD 117	-	-	-	-	-	-	-	-	-	-	17.5	MR	neg	neg	neg	neg	neg	neg	neg
CD 119	-	-	-	-	-	-	-	-	-	-	40	I-MS	neg	neg	neg	neg	neg	neg	neg
CD 121	-	-	-	-	-	-	-	-	-	-	1	R	NSD	neg	H	neg	neg	neg	neg
CD 122	-	-	-	-	-	-	-	-	-	-	7.5	MR-R	NSD	neg	H	neg	neg	neg	neg
CD 123	-	-	-	-	-	-	-	-	-	-	8	MR-R	neg	neg	neg	neg	neg	neg	neg
CD 150	-	-	-	-	-	-	-	-	-	-	37.5	I-MS	neg	neg	neg	neg	neg	neg	neg
CD 151	-	-	-	-	-	-	-	-	-	-	35	I	neg	neg	neg	neg	neg	neg	neg
CD 154	-	-	-	-	-	-	-	-	-	-	55	MS-I	pos	neg	neg	neg	neg	neg	neg
CD 1550	0	0	0	0	0	0	0	1	0	3-	45	MS-S	neg	neg	neg	neg	neg	neg	neg
Celebra	12-	1	22+	12-	1-	;1-	1-	1–1	0	4	5	I	neg	neg	H	neg	neg	neg	neg
FPS Nitron	0	0	0	1-	0	1-	1-	1-	0	0	22.5	I-MR	neg	H	neg	neg	neg	neg	neg
Fundacep 300	-	-	-	-	-	-	-	-	-	-	25.5	S-R	neg	neg	neg	neg	neg	neg	pos
Fundacep 51	-	-	-	-	-	-	-	-	-	-	25	I-MS	neg	neg	neg	neg	neg	neg	pos
Fundacep 52	-	-	-	-	-	-	-	-	-	-	30	I-MS	neg	neg	neg	neg	neg	neg	pos
Fundacep Bravo	-	-	-	-	-	-	-	-	-	-	10	MR	neg	neg	neg	neg	neg	neg	H
Fundacep Campo Real	-	-	-	-	-	-	-	-	-	-	20	I	neg	neg	neg	neg	neg	neg	neg
Fundacep Cristalino	;1-	0; /2		3–3	0	0	1-	1–1/3–3	1-	1–1	90	S	pos	neg	neg	neg	neg	neg	neg
Fundacep Horizonte	-	-	-	-	-	-	-	-	-	-	40	MS	neg	neg	neg	neg	neg	neg	neg
Fundacep Nova Era	-	-	-	-	-	-	-	-	-	-	10	I	neg	neg	neg	neg	neg	neg	pos
Fundacep Raizes	-	-	-	-	-	-	-	-	-	-	37.5	I	neg	neg	neg	neg	neg	neg	neg
IPR 128	-	-	-	-	-	-	-	-	-	-	32.5	I-MS	neg	neg	neg	neg	neg	neg	neg
IPR 130	-	-	-	-	-	-	-	-	-	-	7.5	R-MR	neg	neg	neg	neg	neg	neg	neg
IPR 136	-	-	-	-	-	-	-	-	-	-	50	MS	NSD	neg	neg	neg	neg	neg	neg
IPR 144	-	-	-	-	-	-	-	-	-	-	7.5	MR	H	neg	neg	neg	neg	neg	neg
IPR 85	0	1-	1-	0	;1-	;1-	1	0	;1-	34	13.75	MR	neg	neg	neg	neg	neg	neg	neg
IPR Catuara	0	3-	;1-	1	0	12/3-	12-	0	;1-	33+	67.5	MS-S	neg	neg	neg	neg	neg	neg	neg
Jadeíte 11	;1-	1-	1-	1	0	;1-	1–1	1–1	1	1-	40	I-MS	neg	neg	neg	neg	neg	neg	neg
LG Oro	0	1-	;1-	1-	1-	1-	11+	1-	11+	1–1	65	MS-S	neg	neg	neg	neg	neg	neg	neg
LG Prisma	0	0	1-	0	;1-	1-	0	0	1-	1-	25	S	NSD	pos	neg	neg	neg	neg	neg
Marfim	;1-	1-	0	;1-	;1-	1-	1-	;1-	1-	1–1	15	MRMS	neg	neg	neg	neg	neg	neg	neg
Mirante	0	1–1	0	0	0	1-	1-	1-	1	34	55	MS-S	neg	neg	neg	neg	neg	neg	neg
Onix	-	-	-	-	-	-	-	-	-	-	37.5	MS-MR	neg	neg	neg	neg	neg	neg	neg
OR 1	-	-	-	-	-	-	-	-	-	-	17.5	I	neg	neg	neg	neg	neg	neg	neg
ORS 1401		;1-	0	1-	1-	;1-	;1-	;1-	1-	1-	5.5	MR-MS	neg	neg	pos	neg	neg	neg	neg
ORS 1403	0	0	0	1-	0	1-	;1-	0	0	;1-	1	R	neg	neg	pos	neg	neg	neg	neg
ORS 1405	0	1-	0	1-	0	1-	;1-	1-	0	1-	22.5	I	neg	neg	NSD	neg	neg	neg	neg
ORS Vintecinco	0	1-	1-	1-	;1-	1-	1-	0	1-	1-	10	MR	neg	neg	neg	neg	neg	neg	neg
Pampeano	-	-	-	-	-	-	-	-	-	-	12.5	MR	neg	neg	neg	neg	neg	neg	neg
Quartzo	0	1-	0	1-	1-	;1-	1-	1-	1-	1-	21.3	MR-I	neg	neg	neg	neg	neg	neg	neg
Safira	0	1-	0	1-	0	;1-	1-	0	0	1–1	18.8	MR-I	neg	neg	neg	neg	neg	neg	neg
Supera	1–1	1-	1-	3-	2–2	12-	22-	;1-	1	33+	32.5	I-MS	neg	neg	neg	neg	neg	neg	neg
Tbio Bandeirantes	0	1-	;1-	12-	0	1-	1–1	0	;1-	33+	65	MS-S	neg	neg	NSD	neg	neg	neg	neg
Tbio Iguaçu	1-	1-	1-	1-	1-	;1-	0	0	1-	1-	25	MR-I	neg	neg	NSD	neg	neg	neg	neg
Tbio Itaipu	0	1-	;1-	1-	1-	;1-	1-	1-	1-	1-	20	MR	neg	neg	NSD	neg	neg	neg	neg
Tbio Mestre	0	1–1	0	1-	0	1-	0	0	0	;1	1	R	neg	neg	NSD	neg	neg	neg	neg
Tbio Noble	1	1-	3-	1–1	0	1-	1-	1-	1	34	35	MS	neg	neg	neg	neg	neg	neg	neg
Tbio Pioneiro	11+	1-	2-	1-	0	1-	1-	1-	1–1	4	30	I-R	neg	neg	H	neg	neg	neg	neg
Tbio Seleto		1-	;1-	1-	0	;1-	1-	0	;1-	0	3	R-MR	neg	pos	neg	neg	neg	neg	neg
Tbio Sintonia	1-	1-	22+	1–1	0	;1-	;1-	;1-	0	4	7.5	MR-MS	neg	neg	NSD	neg	neg	neg	neg
Tbio Sinuelo	0	1-	1-	1-	1-	1-	;1-	0	1-	;1-	20	MR-I	neg	neg	neg	neg	neg	neg	neg
Tbio Tibagi	0	1-	0	0	1-	1-	1–1	;1-	1–1	34	10	R-I	neg	neg	neg	neg	neg	neg	neg
Tbio Toruk	1-	1-	1–1	3-	33+	3–3	33-	1-	11+	34	17.5	S-MS	neg	neg	neg	neg	neg	neg	neg
TEC Veloce	-	-	-	-	-	-	-	-	-	-	5	R	neg	neg	neg	neg	neg	neg	neg
TEC Vigore	-	-	-	-	-	-	-	-	-	-	20	S-S	pos	neg	neg	neg	neg	neg	neg
Topazio	;1-	1-	0	1-	0	;1-	1-	1-	0	;1-	11.3	I-R	neg	neg	neg	neg	neg	neg	neg
Turqueza	-	-	-	-	-	-	-	-	-	-	12.5	I-MR	neg	neg	neg	neg	neg	neg	neg
Valente	-	-	-	-	-	-	-	-	-	-	15	S-I	neg	H	neg	neg	neg	neg	neg
Vaqueano	-	-	-	-	-	-	-	-	-	-	7.5	R-MR	neg	neg	neg	neg	neg	neg	neg

^a^ Morden 2017–2018 Field, Canada.

**TABLE 5:** Comparison of FHB reaction in ancient and more contemporary spring wheat Brazilian cultivars in Canada (mean of FHB index and DON, Ottawa 2021 and Morden 2017 and 2018 under artificial inoculation and irrigation) with the classification to FHB in Brazil (Dotto et al., 2005; Informações técnicas para trigo e triticale - Safra, 2012, 2011; Cunha and Caierão, 2014; Cunha et al., 2016; Silva et al., 2017; Franco and Evangelista, 2018; Informações técnicas para trigo e triticale - Safra, 2019, 2018; Informações técnicas para trigo e triticale - Safra, 2020, 2020; Informações técnicas para trigo e triticale - Safra, 2022, 2022). Prediction of the resistance genes in the collection by genotypage of genes/loci related with FHB and plant height (*Rht* genes). Markers related with resistance genes, positive allele (pos) means presence of the resistant allele and negative (neg) represents the susceptible allele. For the *Rht* genes, positive represents the mutant allele (semi-dwarf) and negative is the wild-type. Hetero: heterozygosity in the locus; NSD: not sufficient data; R: resistance; MR: moderate resistance; MS: moderate susceptibility; S: susceptibility.

Cultivar	FHB index (%)	DON (ppm)	FHB Brazil^a^	*Fhb1*	*Fhb2*	*Fhb 5AS*	*FHB 3AL*	*Rht-B1*	*Rht-D1*	*Rht8*
Abalone	18	15	MR MS	neg	neg	neg	pos	pos	neg	neg
Ametista	10.3	9.2	MS S	neg	neg	NSD	NSD	pos	neg	neg
Berilo	32.6	24.5	MS	neg	neg	neg	pos	pos	neg	NSD
BR 18 - Terena	39.6	28.7	S	neg	neg	neg	pos	neg	pos	neg
BR 23	24	20.5	S	neg	neg	neg	NSD	neg	pos	neg
BRS 177	12.1	7.6	MR	neg	neg	NSD	pos	pos	neg	neg
BRS 179	22.2	15.4	MR	neg	neg	pos	pos	neg	neg	neg
BRS 208	24	14.7	MS	neg	neg	neg	NSD	neg	neg	neg
BRS 220	30.8	26.8	MS	neg	neg	neg	NSD	neg	pos	NSD
BRS 254	33	29.9	S	neg	neg	NSD	NSD	pos	neg	neg
BRS 264	38.8	26.8	S	neg	neg	neg	pos	pos	neg	neg
BRS 276	24.6	16.9	MS	neg	neg	neg	pos	pos	neg	neg
BRS 296	21.2	17	MR	neg	neg	pos	neg	pos	neg	neg
BRS 327	20.4	17.4	MR	neg	neg	neg	NSD	neg	neg	neg
BRS 328	22	17.9	MS	neg	neg	pos	NSD	pos	neg	neg
BRS 331	17.6	17.8	MS	neg	neg	neg	NSD	neg	pos	NSD
BRS 374	19.5	24	S	neg	neg	neg	NSD	neg	pos	NSD
BRS Albatroz	18.9	20.1	MS	neg	neg	neg	NSD	pos	neg	neg
BRS Buriti	17.6	8.8	MS	neg	neg	neg	NSD	neg	neg	neg
BRS Camboatá	28.5	22.2	MS	neg	neg	neg	NSD	pos	neg	NSD
BRS Gaivota	23.7	15.6	MS	neg	neg	NSD	NSD	pos	neg	NSD
BRS Gralha Azul	26.8	20.2	MS	neg	neg	NSD	NSD	pos	neg	neg
BRS Guabiju	18.6	17	MS	neg	neg	neg	NSD	neg	neg	neg
BRS Guamirim	20.2	18.7	MR	neg	neg	neg	NSD	neg	pos	neg
BRS Louro	17.2	9.3	MR	neg	neg	neg	pos	pos	neg	neg
BRS Pardela	33.1	23.6	MS	neg	neg	neg	NSD	neg	pos	neg
BRS Parrudo	21.2	14.5	MR	neg	neg	pos	NSD	neg	pos	neg
BRS Sabia	24.1	12.4	MS	neg	neg	NSD	NSD	pos	neg	NSD
BRS Tangará	30.7	31.8	MS	neg	neg	neg	NSD	neg	pos	neg
BRS Timbaúva	17.8	14	MR	neg	neg	neg	pos	pos	neg	NSD
BRS Umbu	-	-	MR	NSD	NSD	neg	NSD	pos	neg	neg
Campeiro	14.6	8.9	MS	neg	neg	neg	NSD	pos	neg	neg
CD 104	24.5	20.6	S	neg	NSD	NSD	pos	pos	neg	pos
CD 105	31.6	27.9	S	neg	NSD	neg	pos	pos	neg	neg
CD 108	32.1	24.4	S	neg	neg	neg	NSD	pos	neg	neg
CD 1104	26.4	16.5	MS	neg	neg	NSD	neg	pos	neg	NSD
CD 113	26.6	29.1	S	neg	neg	neg	NSD	pos	neg	neg
CD 114	21.9	11.9	MS	neg	NSD	neg	NSD	neg	pos	pos
CD 115	17.4	11.4	MS	neg	neg	neg	pos	pos	neg	neg
CD 116	24.8	25.9	S	neg	neg	pos	NSD	pos	neg	neg
CD 117	18.7	20.3	MS	neg	neg	NSD	NSD	pos	neg	hetero
CD 118	24.6	17.4	S	neg	NSD	neg	NSD	pos	neg	NSD
CD 119	41.1	24.9	MS	neg	neg	neg	NSD	pos	neg	NSD
CD 120	-	-	MS	neg	neg	neg	NSD	pos	neg	neg
CD 121	23.5	17.6	MS	neg	neg	NSD	pos	pos	neg	NSD
CD 122	36.6	32.8	MS	neg	neg	NSD	pos	neg	pos	neg
CD 123	25.7	20.4	MS	neg	neg	neg	pos	pos	neg	neg
CD 1252	-	-	S	neg	neg	NSD	NSD	pos	neg	NSD
CD 150	31.7	20.7	S	neg	NSD	pos	pos	pos	neg	hetero
CD 151	25.6	25.7	MS	neg	NSD	pos	pos	pos	neg	hetero
CD 154	25.4	30.6	S	neg	neg	neg	pos	pos	neg	NSD
CD 1550	16.1	13.8	MS	neg	neg	NSD	NSD	pos	neg	NSD
Celebra	17.1	10.5	MR MS	neg	neg	NSD	NSD	pos	neg	NSD
Embrapa 22	39.5	38.5	-	neg	NSD	pos	NSD	pos	neg	neg
Embrapa 42	38.2	34.5	-	neg	NSD	NSD	NSD	neg	pos	neg
FPS Nitron	13.3	8.9	MR MS	neg	neg	NSD	neg	pos	neg	NSD
Fundacep 300	13.7	4.7	S	neg	neg	neg	NSD	hetero	neg	neg
Fundacep 51	11.4	6.8	MS	neg	neg	neg	NSD	neg	neg	neg
Fundacep 52	17.4	10	S	neg	neg	pos	NSD	neg	pos	neg
Fundacep Bravo	16.2	8.1	MS	neg	neg	pos	NSD	pos	neg	neg
Fundacep Campo Real	14.7	9.3	MR	neg	neg	neg	NSD	pos	neg	NSD
Fundacep Cristalino	23.9	15.1	MS	neg	neg	neg	pos	pos	neg	NSD
Fundacep Horizonte	10.5	9.3	MR MS	neg	neg	neg	pos	pos	neg	neg
Fundacep Nova Era	16.8	6.6	S	neg	neg	neg	NSD	neg	pos	neg
Fundacep Raizes	15.7	10.1	S	neg	neg	neg	NSD	hetero	neg	neg
IPR 128	30.6	28.1	S	neg	NSD	neg	NSD	pos	neg	neg
IPR 130	30.4	20.2	S	neg	NSD	neg	NSD	pos	neg	neg
IPR 136	23.8	20.9	S	neg	NSD	pos	NSD	pos	neg	neg
IPR 144	41.5	42.9	S	neg	NSD	NSD	NSD	pos	neg	neg
IPR 85	45.5	32.3	MS	neg	neg	neg	pos	neg	pos	neg
IPR Catuara TM	30.3	18.4	S	neg	neg	neg	pos	neg	pos	neg
Jadeite 11	12	7.5	MR	neg	neg	NSD	neg	pos	neg	NSD
LG Oro	12.7	12.9	MS	neg	neg	NSD	NSD	pos	neg	NSD
LG Prisma	17.9	11.8	MR	neg	neg	NSD	pos	NSD	NSD	NSD
Marfim	21.4	16.1	MS S	neg	neg	neg	NSD	pos	neg	NSD
Mirante	17.5	10.6	S	neg	NSD	neg	NSD	pos	neg	neg
Onix	17	6.7	MS	neg	neg	neg	NSD	pos	neg	neg
OR 1	20.9	14.8	MS	neg	NSD	neg	NSD	pos	neg	neg
ORS 1401	12.5	10.3	MR	neg	neg	NSD	pos	pos	neg	NSD
ORS 1402	-	-	MR	neg	neg	NSD	NSD	NSD	neg	NSD
ORS 1403	27.8	7.2	MR	neg	neg	NSD	NSD	pos	neg	NSD
ORS 1405	14.4	5.1	MR MS	neg	neg	NSD	neg	pos	neg	NSD
ORS Vintecinco	16.6	12.9	-	neg	neg	NSD	NSD	pos	neg	NSD
Pampeano	17.9	8.9	MR	neg	neg	neg	pos	pos	neg	neg
Quartzo	16.1	12.2	MS	neg	NSD	NSD	NSD	pos	neg	NSD
Safira	25	11.6	MS	neg	neg	neg	NSD	pos	neg	neg
Supera	23.4	21.1	MS	neg	NSD	neg	NSD	pos	neg	neg
TBio Bandeirante	20.1	18.4	MS	neg	neg	NSD	NSD	pos	neg	NSD
TBio Iguaçu	17.4	8.8	MS	neg	neg	NSD	NSD	pos	neg	NSD
TBio Itaipu	17.4	10.9	MS	neg	neg	NSD	NSD	pos	neg	NSD
TBio Mestre	18.7	15.7	MS	neg	neg	NSD	pos	pos	neg	NSD
TBio Noble	21.7	14.3	MS	neg	neg	NSD	neg	NSD	NSD	NSD
TBio Pioneiro	16.9	10.4	MS	neg	neg	NSD	neg	pos	neg	NSD
TBio Seleto	13.3	6.9	MS MR	neg	neg	NSD	NSD	pos	neg	NSD
TBio Sintonia	22.7	13.4	MS	neg	neg	NSD	NSD	pos	neg	NSD
TBio Sinuelo	13.7	6.3	MS MR	neg	neg	NSD	NSD	pos	neg	NSD
TBio Tibagi	22.9	12.7	S MS	neg	neg	NSD	NSD	pos	neg	NSD
TBio Toruk	14.5	11.3	S MS	neg	neg	NSD	NSD	pos	neg	NSD
TEC Frontale	-	-	-	neg	neg	NSD	NSD	pos	neg	NSD
TEC Triunfo	-	-	MR MS	neg	neg	NSD	NSD	NSD	NSD	neg
TEC Veloce	18.7	12	MR MS	neg	neg	neg	pos	pos	neg	NSD
TEC Vigore	11.8	5.8	MR MS	neg	neg	neg	pos	pos	neg	NSD
Topazio	16.8	11.6	MR	neg	neg	neg	NSD	pos	neg	neg
Turqueza	14.3	11.1	MR	neg	neg	neg	neg	pos	neg	neg
Valente	23.1	14.6	S	neg	neg	neg	NSD	pos	neg	neg
Vaqueano	18.7	13.7	MS MR	neg	neg	neg	NSD	pos	neg	neg

^a^ Informações Técnicas para Trigo e Triticale, disease reaction provided by cultivar’s sponsor.

Only collection “B” was tested at seedling stage for their reaction to nine Canadian *P. graminis* f. sp. *tritici (Pgt)* races and the Ug99 race “TTKSK” (Table 4). Of the 42 cultivars tested 37 were resistant to the *Pgt* races, and 28 cultivars showed seedling resistance to the Ug99 stem rust race TTKSK. Both collections were field evaluated at Morden (MB) in 2017 and 2018 with severity ranging from 1R to 90S (Table 3; Figure 2). ORS 1403 demonstrated excellent stem rust resistance. This cultivar demonstrated seedling resistant to all 9 Canadian races and TTKSK, and it was rated 1R in the field. Molecular analyses indicated that ORS 1403 carries the 2NS translocation (*Sr38*), but does not have the DNA markers for *Sr31* (1RS:1BL), *Sr57*, *Sr23*, *SrCad*, or *Sr2*. The rye translocation (1RS:1BL), which has been associated with leaf, stem and stripe rust and powdery mildew resistance, was present in 14% of the cultivars (Figure 6; Table 4).

When tested for stripe rust resistance, 46% of the Brazilian cultivars had average severity equal to or less than 30% in 2015 and 2017 (Figure 2). In general, the Brazilian material possessed less stripe rust resistance compared to leaf rust resistance. 26% of Brazilian cultivars tested had stripe rust severity over 50%.

The powdery mildew nurseries in 2017 and 2018 had maximum plot severities of 70% and 60%, respectively. Among Brazilian cultivars evaluated, 53% had powdery mildew severity scores less than or equal to 20% (Figure 2).

Figure 2 demonstrates the reaction of each cultivar to leaf rust, stripe rust, stem rust and powdery mildew. Four cultivars were found to possess superior resistance to the three rusts and powdery mildew: ORS 1403, CD 121, BRS Camboata and Tbio Mestre.

### 3.3 Fusarium head blight

**FIGURE 3:**
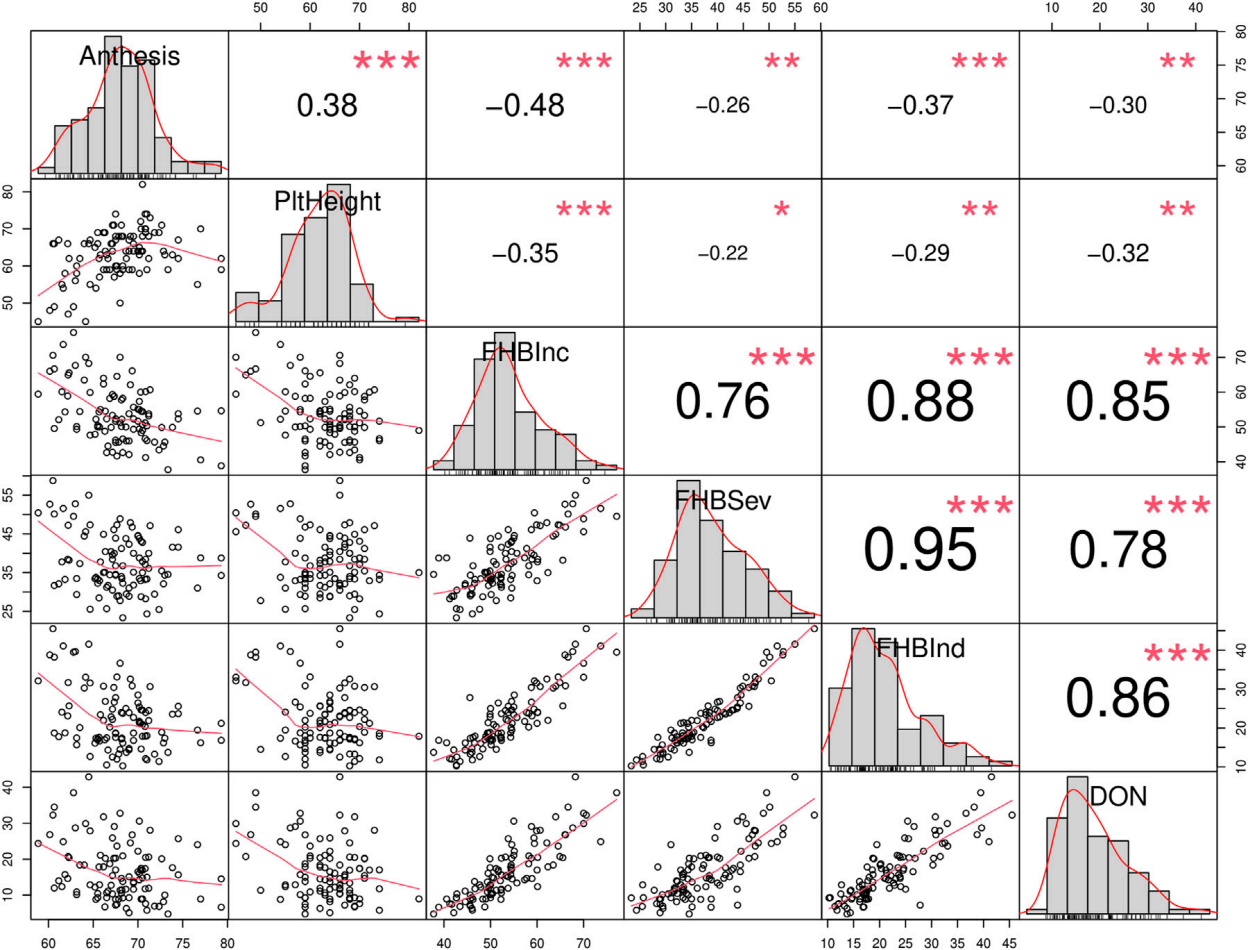
Correlation matrix of traits related with Fusarium head blight (FHB): Anthesis (days), Plant height (PltHeight, cm), incidence (FHBInc, %), severity (FHBSev, %), index (FHBInd, %) and Deoxynivalenol (DON, ppm). The distribution of each variable is shown on the diagonal. On the bottom of the diagonal, the bivariate scatter plots with a fitted line are displayed. On the top of the diagonal, the value of the correlation plus the significance level as stars. Each significance level is associated to a symbol: 0.001: “***”, 0.01: “**”, 0.05: “*”. The data is derived from the evaluation of Brazilian cultivars grew at Ottawa 2021 and at Morden 2017 and 2018 under artificial inoculation and irrigation.

Results of the Brazilian collections at the FHB nurseries at Morden 2017, 2018 and Ottawa 2021 showed significant positive correlations among the traits associated with FHB disease rating (Incidence, Severity, FHB Index and DON). These disease rating parameters were negatively correlated with anthesis and plant height (Figure 3).

**FIGURE 4:**
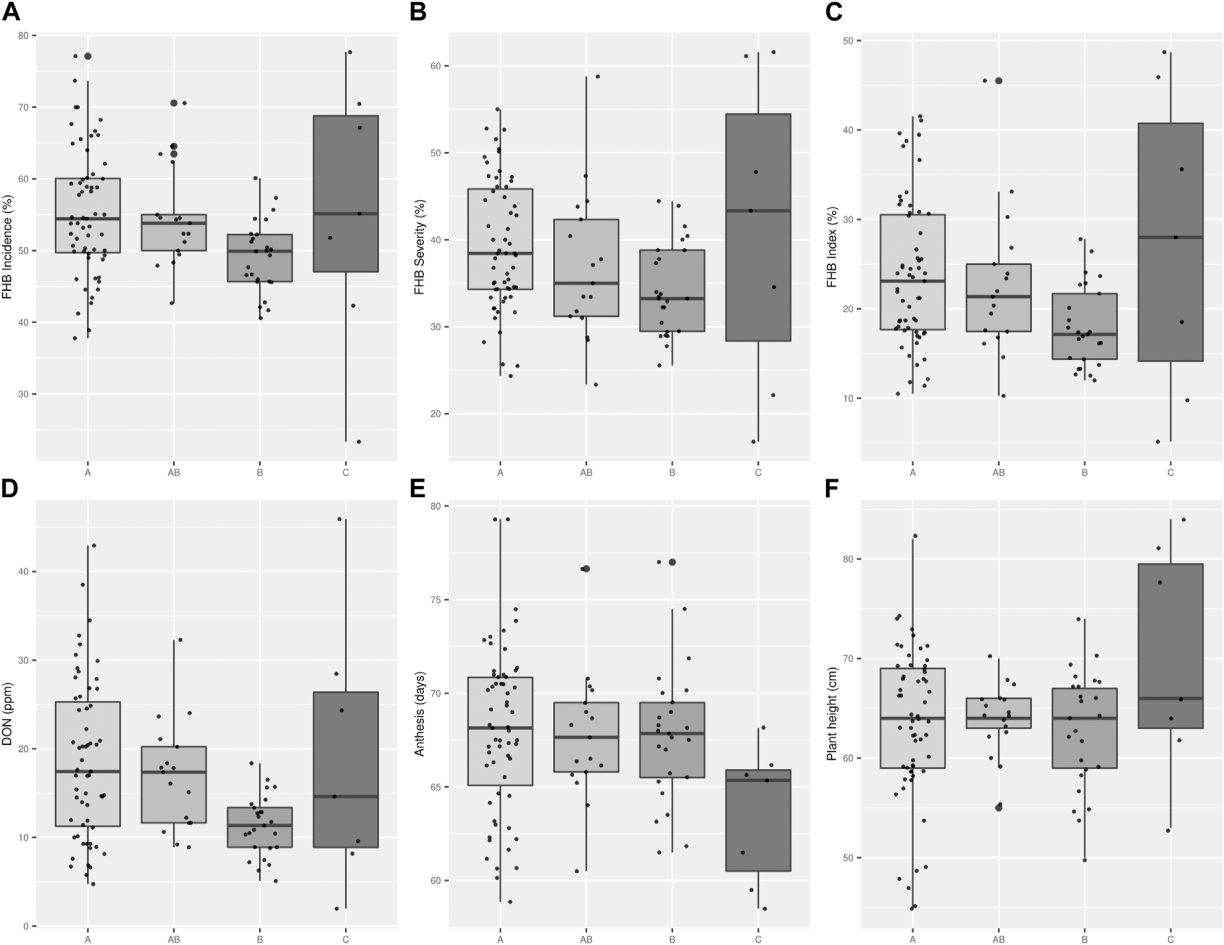
Boxplot charts representing the distribution frequency of the analysed Brazilian cultivars and Canadian checks in traits related with Fusarium head blight (FHB): incidence **(A)**, severity **(B)**, index **(C)**, Deoxynivalenol (DON **(D)**), anthesis **(E)** and plant height **(F)**. The Brazilian cultivars were divided by collections: “A” present in the older collection, “B” present in the newer collection, “AB” in both collections. “C” refers to Canadian checks. The data is derived from the evaluation of Brazilian cultivars grew at Ottawa 2021 and at Morden 2017 and 2018 under artificial inoculation and irrigation.

**FIGURE 5:**
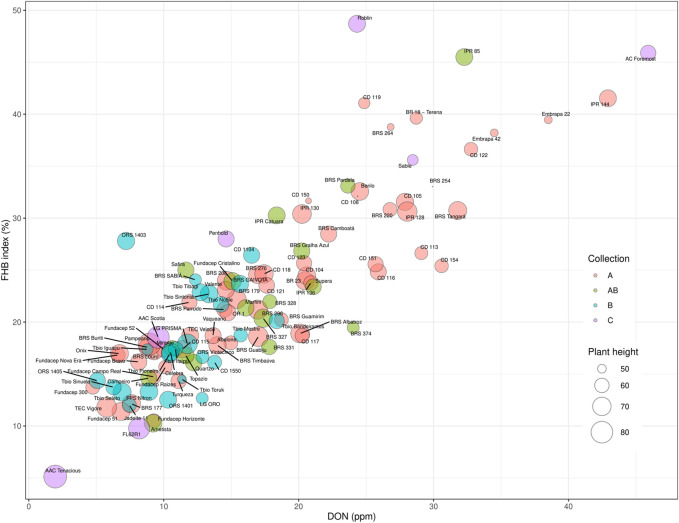
Each analysed cultivar plotted in a bubble plot with FHB index as y-axis, DON accumulation at x-axis and plant height—size of the bubble. The cultivars were classified by the collections, which created a fourth dimension (color) in the plot: “A” red, “B” blue, “AB” green, “C” purple, which refers to Canadian checks.

When the FHB ratings and DON data were considered by collection, there was a trend to lower FHB disease scores (Incidence, Severity, FHB index, and DON) from collection ‘A’ (older cultivars), to cultivars in collection ‘B’ (recent cultivars) (Figures 4A–D). Improved FHB resistance of collection B is also evident in Figure 5, where these cultivars were mostly situated in the bottom left quadrant of the chart indicating a FHB index below 30% and DON content below 20 ppm. In general, Canadian checks were earlier maturing than Brazilian cultivars, with some exceptions (Figure 4E). While there was a significant negative correlation between both FHB index and DON with plant height, some shorter Brazilian cultivars with good FHB resistance were identified (Table 3; e.g., Fundacep 300, Fundacep Horizonte, Jadeite and LG Oro).

**FIGURE 6:**
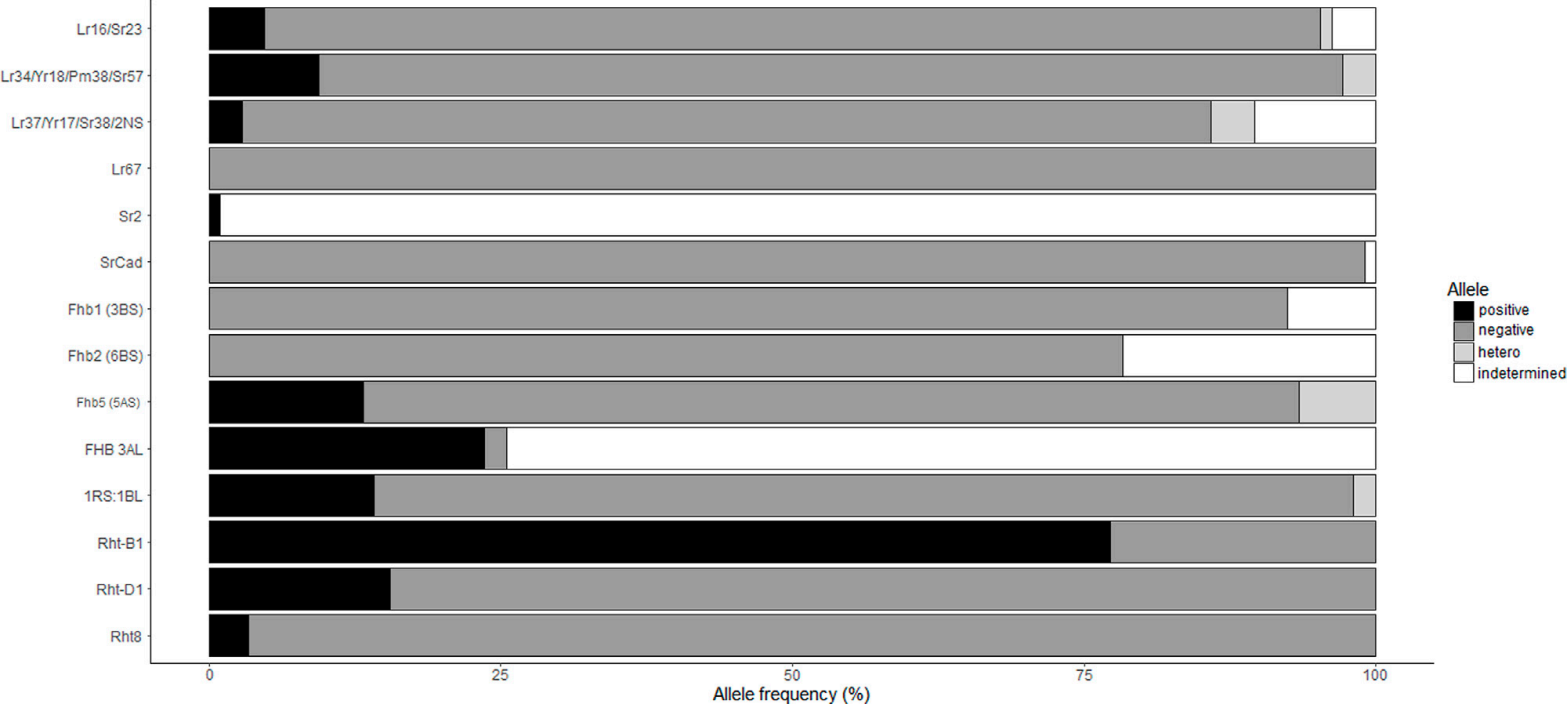
Alleles frequency in the Brazilian collection. The Brazilian cultivars (106, except *Rht8*—60 cultivars, *Rht-B1*—101 cultivars and *Rht-D1*—103 cultivars) were genotyped with molecular markers related with genes/loci of traits of interest. Each material was classified as positive (colored as black), negative (dark grey), heterozygous (light grey) or indetermined (null or questionable allele, white). Markers related with resistance genes, positive allele means presence of the resistant allele and negative represents the susceptible allele. For the *1RS:1BL*, positive allele represents presence of the translocation. For all others, positive represents the mutant allele and negative is the wild-type.

FHB resistance within the collections does not appear to be conferred by *Fhb1* or *Fhb2*, because cultivars had the susceptible alleles for markers close to those loci (Table 5; Figure 6). In contrast, FHB QTL *3AL*, derived from Frontana, was identified in 24% of the cultivars tested, and 13% of the cultivars tested positive for resistant alleles associated with 5AS FHB QTL. The KASP marker IWA7777 developed for the 5AS FHB QTL (Pandrurangan et al., 2021) gave positive alleles for both Frontana and Sumai-3. Thus, it is not possible to distinguish the source of this QTL using IWA7777 marker. For SSR marker *gwm293*, Frontana (211bp) and Sumai-3 (217bp) amplified different size alleles. The cultivars that amplified the same *gwm293* allele as Frontana for FHB 5AS were: Ametista (heterozygous), BRS179, BRS254, BRS 296, BRS 328, BRS Parrudo, CD 150, CD 151, Embrapa 22, Embrapa 42, IPR 136, TEC Triunfo; and the cultivars that amplified same allele as Sumai-3 for FHB 5AS (*gwm293*) were: CD 116, CD 121, Fundacep 52, Fundacep Bravo. Four cultivars had the alleles associated with FHB resistance for the QTL on 5AS and 3AL: BRS 179, CD 121, CD 150 and CD 151 (Table 5). The comparison of the cultivars with the allele associated with FHB resistance at *FHB 5AS* QTL with cultivars with the susceptible allele found no significative difference among the two groups for FHB index (*p* = 0.89) and DON level (*p* = 0.69) (data not shown).

**FIGURE 7:**
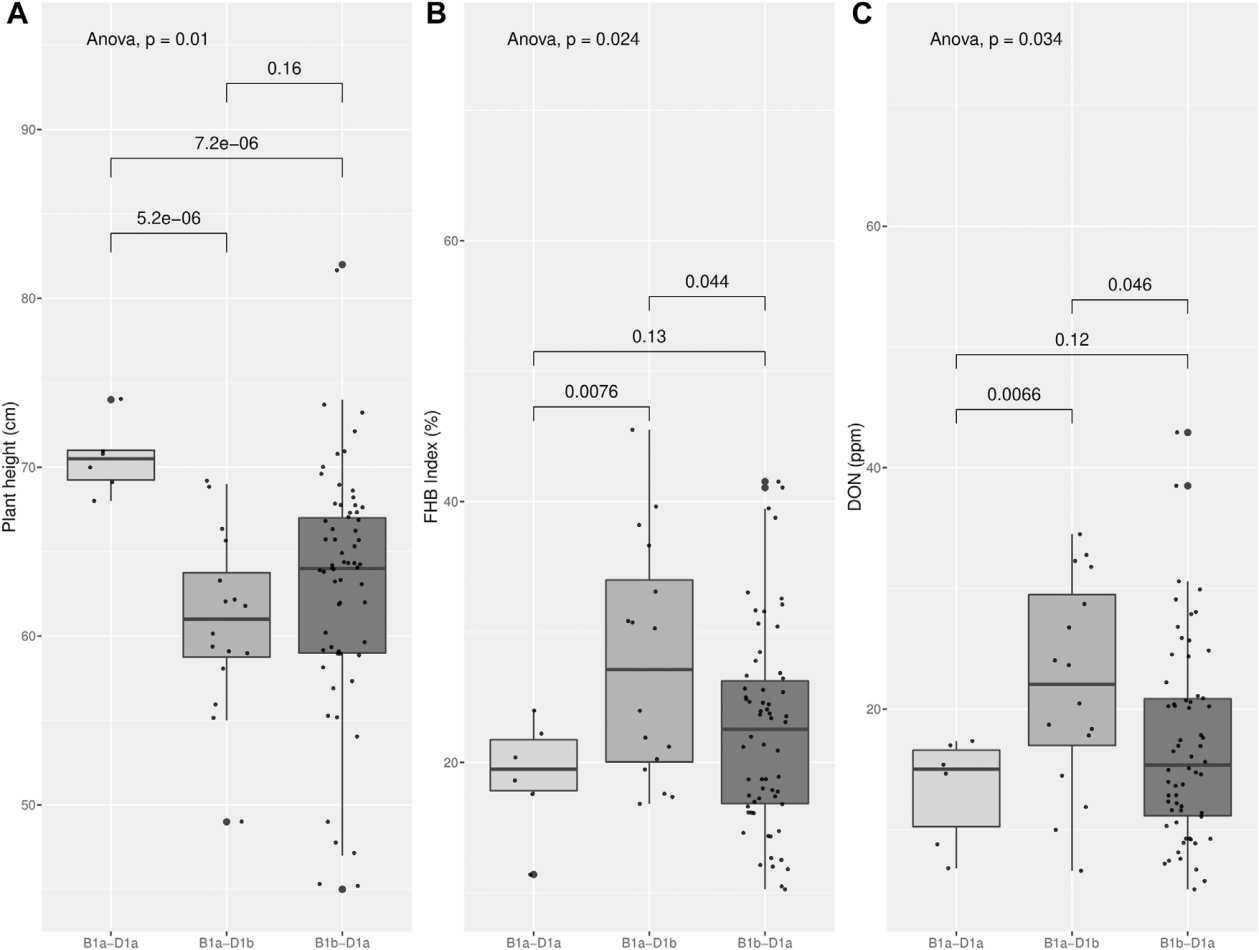
Plant height **(A)**, FHB index **(B)** and DON accumulation **(C)** in the wheat collections tested at Morden in 2017 and 2018 classified by the presence (“a” haplotype) or absence (“b” haplotype) of the mutation at *Rht-B1* and *Rht-D1* genes predicted by molecular markers. Effect of each class *B1a- D1a*, *B1a-D1b* and *B1b-D1a* in those traits and comparison of the means of each paired class (t-test).

Through molecular marker analysis, the presence of the semi-dwarf alleles, *Rht-B1b* and *Rht-D1b,* was predicted. The semi-dwarf *Rht-B1b* allele was present in 77.2% of the cultivars tested compared to only 15.5% of cultivars that carried the *Rht-D1b* allele (Figure 6). Only six cultivars did not carry either the *Rht-B1b* or the *Rht-D1b* semi-dwarf alleles (Table 5), of which two cultivars (CD104 and CD114) tested positive for the *Rht8* semi-dwarf allele. These two Brazilian cultivars may have either Akakomugi or Strampelli in their ancestry because the *Rht8* marker is only diagnostic in germplasm with these exotic cultivars in their lineage (Ellis et al., 2007). The effect of the *Rht* genes on plant height and FHB resistance is shown in Figure 7. T-tests indicated significant differences in plant height between the *B1a-D1a* cultivars and cultivars with either the *Rht-B1b* or the *Rht-D1b* allele. In the presence of the *Rht-D1b* allele, FHB symptoms and DON accumulation were higher compared to cultivars without it. The presence of the *Rht-B1b* allele, on average, did not significantly affect the FHB symptoms and DON accumulation in the cultivars tested (Figure 7).

## 4 Discussion

Wheat cultivars from both countries have been developed with good performance in their agroclimatic conditions and good levels of resistance to important economic diseases. In this study, we evaluated the agronomic performance Brazilian cultivars under Canadian growing conditions, we assessed the reaction to Canadian isolates/pathogens and hypothesize as to the presence of certain genes. The final aim was to understand the weakness and strengths of the Brazilian germplasm in order to effectively use it with Canadian genetics to increase genetic diversity, improve genetic gain and resilience in spring wheat.

In general, the Brazilian spring wheat collection with more recently released cultivars, had intermediate protein and higher grain yield compared to the older collection (Figure 1). The well-known negative correlation between yield and protein was evident in 2018 but the yield-protein content relationship was not significant in 2017. Most Brazilian cultivars tested that plotted below the linear regression line of yield *versus* protein (Figure 1), were removed from the 2022 Brazilian recommendation list of cultivars (Supplementary Table S1). The exceptions were Marfim (hard red wheat with white flour) and Campeiro (hard red wheat with crackers-flour type), which have specific end-use quality attributes.

The genetic basis for disease resistance can be a determining factor in the durability of disease resistance. Frequently, rust resistance genes that were previously effective at controlling the disease, lose their effectiveness due to changes in the pathogen population, resulting in susceptibility. Recent examples are the evolution of virulence to the leaf rust resistance gene *Lr21* which conditioned complete resistance in Canada until the emergence of virulent races in 2012 (McCallum et al., 2018), or *Sr31* and/or *Sr24* stem rust resistance genes that were overcome by Ug99 isolates and variants in Africa (Fetch et al., 2021). However, disease resistance has never been overcome for the durable multi-pest resistance genes *Lr34* and *Lr46* (Bokore et al., 2022). To avoid resistance genes becoming ineffective, it is important to diversify the genetics and pyramidize genes, using durable adult plant resistance genes and/or minor genes.

Wheat breeders have been able to achieve high levels of resistance for leaf rust in both countries. In Canada, some of the commonly deployed leaf rust resistance genes include *Lr1*, *Lr2a*, *Lr13*, *Lr14a*, *Lr16*, *Lr21*, *Lr34*, and more recently *Lr46* (McCallum et al., 2016; Bokore et al., 2022). The Brazilian wheat cultivars had excellent field and seedling resistance to wheat leaf rust in Canada. However, only a few Brazilian cultivars were positive for the presence of either *Lr34* or *Lr16*, two of the most common resistance genes in Canadian wheat (McCallum et al., 2007; McCallum et al., 2012). Therefore, the Brazilian wheat germplasm likely could be used to diversify and improve the leaf rust resistance of Canadian wheat.

Many Brazilian cultivars tested have good adult plant resistance (APR) because they were seedling susceptible to one or more common Canadian leaf rust races but were at least moderately resistant in the field. In Brazil, these cultivars with APR to leaf rust were classified from MRR to S (Table 3) (Dotto et al., 2005; Informações técnicas para trigo e triticale - Safra, 2012, 2011; Cunha and Caierão, 2014; Cunha et al., 2016; Silva et al., 2017; Franco and Evangelista, 2018; Informações técnicas para trigo e triticale - Safra, 2019, 2018; Informações técnicas para trigo e triticale - Safra, 2020, 2020; Informações técnicas para trigo e triticale - Safra, 2022, 2022). Molecular marker analyses indicate that APR is mostly conferred by genes other than *Lr34* or *Lr67*. *Lr46* may be a source of APR in the Brazilian germplasm or the resistance could be derived from genes not yet identified. Genetic studies to better understand the Brazilian leaf rust resistance are planned with expectations of identification of new sources of durable resistance, such as that identified in the older Brazilian cultivars Toropi and Frontana. The Brazilian cultivar Toropi has excellent adult plant resistance (Barcellos et al., 2000; Rosa et al., 2019) as does Frontana (Dyck and Samborski, 1982; Singh and Rajaram, 1992), which was the original source of *Lr34* resistance complex in the majority of CIMMYT and North American hard red spring wheat cultivars. The genetic resistance of Toropi is complex, involving race-specific and non-specific adult plant genes, being the last denominated as *Lr78* (Barcellos et al., 2000; Wesp-Guterres et al., 2013; Casassola et al., 2015; Kolmer et al., 2018; Rosa et al., 2019).

The common leaf rust differential set used to differentiate and denominate *P. triticina* isolates is inadequate for testing in Brazil (Barcellos and Turra, *pers. comm*.). The seedling differential set used in Brazil to determine the virulence phenotype of the isolates in 2013 was composed by the genes: set 1- *Lr1, Lr2a, Lr2c, Lr3*; set 2- *Lr9, Lr16, Lr24, Lr26*; set 3- *Lr3ka, Lr11, Lr17, Lr30*; set 4- *Lr10, Lr18, Lr21, Lr23*; set 5- *Lr14a, Lr14b, Lr27+Lr31, Lr20*; additional lines *Lr3bg*, ORL 4002 [described by Rosa (2013)]. The seedling gene conferring resistance to ORL 4002 has not been identified. From 2004 to 2007, the predominant race was MDT-MT 4002 avirulent, which was surpassed by the race MDT-MT 4002 virulent. This race was predominant from 2007 to 2010. In 2007, the new races TDT-MT and TFT-MT were identified, and TFT-MT represented approximately 70% of the isolates from 2013 to 2017. Since 2019, variants of TFT and MDT were identified: MPP-MT, TFT-HT, TPT-MT, TNT-MT, TDT-HT. In Canada, the predominant race in 2019 was MNPS, identified first in 2015, and it has been the predominant race since 2016. In 2018, TBBG made up 29% of races and was virulent on *Lr21* (McCallum et al., 2021). There are some differences in the fourth set between Brazil and Canada (*LrB, Lr10, Lr14a, Lr18*), which makes comparison difficult. The Canadian MNPS would be MNP-L or MNP-M in Brazil; while TBBG would be denominated TBB-N or TBB-P. In general, the Brazilian leaf rust races are more virulent than in Canada. In 2023, the Brazilian differential set included *Lr51* and *Lr41* (Nogal). The cultivars Citrino (*Lr26* + other gene(s)), Toruk, ORS1403 recently became susceptible to leaf rust in Brazil. However, in Canada, the last two cultivars were still resistant in seedling and field tests (Table 3). Besides the seedling resistance of ORS1403, this cultivar also demonstrated as yet unidentified APR.

Resistance for stem rust was variable within the Brazilian cultivars. Stem rust has not been a disease of economic importance in Brazil for decades. However, susceptible pustules of stem rust were identified in 2022 on wheat grown at southern Brazil. The most important genes conferring stem rust resistance in Brazilian germplasm at the present time are probably *Sr24* and *Sr31* (1BL.1RS translocation) (Barcellos, *pers. comm*.). The wheat-rye translocation, which is related with multiple disease resistance, has been used by Brazilian and North American breeding programs because it can confer higher yield in some environments, however, it is also related with poor bread quality. This translocation was present in 14% of the cultivars (Figure 6; Table 4). Previously, it was estimated that 1BL.1RS was present in 30% of the area cultivated with wheat in Brazil in 2004 (Germán et al., 2007). The difference in prevalence of 1BL.1RS may reflect the choice by Brazilian producers of the higher yielding cultivars that also carry the 1BL.1RS translocation.

Many cultivars had high levels of both seedling and field stem rust resistance to Canadian *Pgt* isolates. Many also had seedling resistance to TTKSK, one of the Ug99 strains from Africa. Twelve cultivars were tested in Kenya in 2015, and some showed good resistance to the African *Pgt* races (Ametista 30MSS, Campeiro 40MSS, Jadeite 30MSS, Marfim 40MSS, Mirante 60MSS, ORS1401 40MSS, ORS1403 20MR, ORS1405 50MSS, ORS Vintecinco 60MSS, Quartzo 60S, Safira 40MSS, Topazio 20M) (Barcellos, unpublished results). The stem rust resistance genes in these Brazilian cultivars might be different from those in Canadian cultivars making them useful for diversifying the base of stem rust resistance in Canadian wheat. Studies to characterize Brazilian germplasm, such as the one reported here, are valuable for both Brazilian and Canadian researchers.

The Brazilian cultivars had considerable variability for both stripe rust and powdery mildew resistance, with many cultivars being susceptible to either one or the other or both in Canada. However, many cultivars had good levels of stripe rust or powdery mildew resistance, and some had resistance to both (Figure 2). Higher susceptibility for stripe rust than leaf rust in the Brazilian germplasm could be explained by the absence of the first disease in Brazil for decades, so breeding for resistance to stripe rust has been practically absent. Few breeding programs have the capacity to use molecular marker selection in Brazil, so the selections are based on the pedigree and phenotyping in the field, which was impossible without the disease. Recently, Brazil and Canada have experienced stripe rust epidemics, even in warmer regions (Rioux et al., 2015). Therefore, a focus will be needed to introduce stripe rust resistance and to characterize the genetics of the germplasm. This study would be helpful, and Canadian wheat might be a useful source of stripe rust resistance in Brazil. In Canada, stripe rust resistance breeding had not been a priority until after 2000 since it was confined mainly to southern Alberta (McCallum et al., 2007). However, with the spread of stripe rust through the Great Plains of the United States and into the Canadian provinces of Manitoba, Saskatchewan and Alberta since 2000, resistance to stripe rust has become a more urgent priority. One major source of resistance initially in Canadian wheat cultivars was *Yr18* = *Lr34* that had been incorporated into many cultivars for leaf rust resistance, but also conferred stripe rust resistance (McCallum et al., 2007). Powdery mildew is an economic important disease in eastern Canada. However, it is normally of minor importance in western Canada where the majority of wheat is grown, due to the generally warmer and drier growing conditions, which are not favorable to the pathogen. Wheat cultivars are selected for resistance in eastern Canada for powdery mildew resistance but not in western Canada. In Brazil, the environmental conditions favor the disease development, and genetic resistance is key to manage it. In general, *Pm4a, Pm4b, Pm3f, Pm8 and Pm17* are effective in southern Brazil (Lau et al., 2020).

Cultivars were identified that combined resistance to FHB, leaf, stem, stripe rust and powdery mildew including: ORS 1403, CD 121, BRS Camboata and Tbio Mestre. This could be due to pyramids of resistance genes specific to each disease and/or the presence of multi-pest resistance genes, although the best-known multi-pest resistance gene *Lr34* was not found in these multi-disease resistant cultivars. The presence of *Lr46*, another important multi-pest resistance gene should be investigated, which should be facilitated once a diagnostic molecular marker is available.

FHB resistance is less well understood, but Canadians and Brazilians have developed cultivars with good FHB resistance. Canadian wheat cultivars were built from a moderate level of resistance in cultivars such as AC Barrie by adding *Fhb1* from Sumai-3 and other Asian sources (Thambugala et al., 2020). In contrast, FHB resistance in Brazilian wheat is not based on Sumai-3, as molecular markers for *Fhb1* and *Fhb2* were absent in the Brazilian material tested. A similar result was reported by Mellers et al. (2020). In a panel of 558 bread wheat accessions maintained by Brazilian Agricultural Research Corporation (EMBRAPA)-Trigo, which represents wheat germplasm released between 1852 and 2013, only two Brazilian cultivars amplified the resistance allele of *Fhb1*, Peladinho and BR-43, released in 1978 and 1991, respectively. Neither cultivar was included in the present study. The FHB resistance in the Brazilian wheat seems to be based on resistance from the Brazilian cultivar “Frontana”. The presence of the 3AL QTL, derived from Frontana, and the 5AS QTL (identified in both Frontana and Sumai-3) was shown, based on molecular marker testing, in 11 and 28 cultivars, respectively. However, a comparison of the FHB index and DON level of cultivars with the positive allele for *5AS* QTL and cultivars with the negative allele indicated no significative difference among the cultivars. Therefore, the effect of *FHB 5AS* QTL alone was not enough to confer resistance. The results of the 3AL QTL marker testing were not adequate to predict the presence/absence of these QTL in many of the cultivars, so similar analysis of the effect of 3AL QTL was not possible.

Cultivars that had the highest FHB index and DON levels in Canada were classified as MS or S in Brazil (Dotto et al., 2005; Informações técnicas para trigo e triticale - Safra, 2012, 2011; Informações técnicas para trigo e triticale - Safra, 2013, 2013; Cunha and Caierão, 2014; Riede, 2014; Cunha et al., 2016; Silva et al., 2017; Franco and Evangelista, 2018; Informações técnicas para trigo e triticale - Safra, 2019, 2018; Informações técnicas para trigo e triticale - Safra, 2020, 2020; Informações técnicas para trigo e triticale - Safra, 2022, 2022). However, some Brazilian cultivars classified as MS or S in Brazil performed well in Canada (Table 5). The climate conditions in Brazil are frequently favorable to the development of *Fusarium* infection, with temperatures over 20°C and high precipitation during anthesis. The inoculum pressure is very high, as the fungi can survive in many native species and previous crops (Chiotta et al., 2021). Wheat is mainly rotated with soybean and corn in southern Brazil, where wheat is predominantly cultivated. In that region, no-till is the predominant farming system.

Besides climate, farming system and inoculum pressure, another difference between Canada and Brazil that could cause the difference in the disease resistance rating of cultivars is the composition of the *F. graminearum* species complex (FGSC). In a multi-year survey of more than 200 wheat fields assessed and more than 600 isolates, the FGSC was composed by *F. graminearum* (83%) of the 15-ADON genotype, *Fusarium meridionale* (12.8%) and *Fusarium asiaticum* (0.4%) of the nivalenol (NIV) genotype, and *Fusarium cortaderiae* (2.5%) and *Fusarium austroamericanum* (0.9%) with either the NIV or the 3-ADON genotype (Del Ponte et al., 2015). In Canada, *F. graminearum sensu stricto* is prevalent (Amarasinghe et al., 2015; Amarasinghe et al., 2019). In some Canadian provinces, the native 15-ADON chemotypes are being displaced by the more aggressive 3-ADON chemotypes, and new NIV-type as well as NX-2 populations have emerged (Kelly et al., 2016; Kelly and Ward, 2018).

The presence of the semi-dwarf alleles was predicted in most of the Brazilian cultivars. Over 75% of the cultivars possessed the semi-dwarf allele of *Rht-B1*, while the *Rht-D1* and *Rht-8* were less frequent (Figure 6). While *Rht* genes reduce plant height, they are also associated with increased susceptibility to FHB (Draeger et al., 2007; Srinivasachary et al., 2008, 2009; Lv et al., 2014). A significant negative correlation between plant height and FHB was detected (Figure 3). The presence of the *Rht-B1b* or *Rht-D1b* alleles significantly reduced plant height although there was no significant difference between the plant height reduction of the two genes. The presence of *Rht-D1b* significantly increase FHB index scores and DON accumulation compared to the *Rht-D1a* allele. This trend was not observed for the *Rht-B1* cultivars (Figure 6). As previously mentioned, Brazilian breeding selections were dependent on phenotyping in the field. Therefore, the *Rht-B1b* was probably indirectly selected due to better FHB resistance in the shorter plants. The success through the years of improving FHB in the Brazilian germplasm is visualized in Figure 4, where most recent cultivars (collection “B”) have lower Fusarium ratings and DON content compared to older cultivars. Selections for reduced plant height and days to anthesis was generally similar across the collections. This study indicates that *Rht-B1* is a preferred option compared to *Rht-D1* to reduce plant height with less effect on FHB susceptibility. A similar result was previously reported for winter wheat in Ontario, Canada (Tamburic-Ilincic and Rosa, 2017; Tamburic-Ilincic and Rosa, 2019). Surprisingly, the frequency of *Rht-B1b* (28%) and *Rht-D1b* (45%) in Southern Great Plains, eastern United States and Canada, are contrary to the proportions reported for the Brazilian collections (Guedira et al., 2010).

Wheat blast (*Magnaporthe oryzae Triticum, MoT*) is a major threat to wheat production in Brazil, other South America and African countries (Li et al., 2015; Callaway, 2016; Tembo et al., 2020; Singh et al., 2021; Hossain, 2022). There are concerns that the pathogen may continue to spread to other parts of the world, including Canada (Kohli et al., 2011; Duveiller et al., 2016; Ceresini et al., 2019). Once the symptoms of blast appear in the spikes, control methods are not efficient and total crop loss can result. Resistance is mostly limited to 2NS carriers, which is being eroded by the newly emerged MoT isolates, demonstrating an urgent need for identification and utilization of non-2NS resistance sources (Singh et al., 2021). The translocated 2NS segment is closely linked to *Sr38, Yr17* and *Lr37* (Bariana and McIntosh, 2011), and some Canadian cultivars have the translocation such as CDC Stanley (Randhawa et al., 2013). Some Brazilian cultivars described in this study could be used as source of 2NS blast resistance for Canadian wheat breeding.

Generally, the Brazilian wheat cultivars performed and yielded well under eastern Canadian growing conditions. Resistance was generally very good for FHB and leaf rust, but more variable between cultivars for stem and stripe rust and powdery mildew. Since the genetic backgrounds of these cultivars are generally different from Canadian wheat, they represent opportunities to improve agronomic performance, and resistance to these diseases. A better understanding of the genetics that condition resistance to each of these diseases will be helpful in parental selection and the development of molecular markers for marker assisted breeding.

## Data Availability

The original contributions presented in the study are included in the article/Supplementary Material, further inquiries can be directed to the corresponding author.
