# Supplementary material for: Characterization of Brazilian spring wheat germplasm and its potential for increasing wheat genetic diversity in Canada
Source: Front Genet. 2023 Mar 17;14:1125940. doi: 10.3389/fgene.2023.1125940 (PMC10063806; doi:10.3389/fgene.2023.1125940)
Supplement: Supplementary file 1 [file Table1.pdf]

Supplementary Material

Characterization of Brazilian spring wheat germplasm and its potential for increasing wheat genetic diversity in Canada

Silvia Barcellos Rosa[1]*, Gavin Humphreys[2], Linda Langille[2], Harvey Voldeng[2], Maria Antonia Henriquez[3], Andrew James Burt[2], Harpinder Singh Randhawa[4], Tom Fetch[5], Colin W. Hiebert[3], Barbara Blackwell[2], Taye Zegeye[3], Allan Cummiskey[6], Eric Fortier[1], Pedro Luiz Scheeren[7], Camila Turra[8], Brent McCallum[3]

*** Correspondence:** Silvia Barcellos Rosa: silvia.rosa@cerom.qc.ca

Supplementary Table 1. Brazilian cultivars classified into collections based on their registration time in Brazil and receipt by AAFC: 'A' - cultivars registered from 1986 to 2012; 'B' - cultivars registered from 1999 to 2016; 'AB' - cultivars present in both collections.

Collection	Cultivar	Pedigree	Launch year	In recommendation to grow in 2022 [1]
A	Abalone	ORL93299/3/ORL92171//EMB16/2*OR 1/4/Rubi	2006	
AB	Ametista	PF 950351/Abalone//Ônix	2011	x
A	Berilo	ORL 99192/ORL 00241	2011	
A	BR 18 - Terena	Alondra Sel.	1986	x
A	BR 23	CC/ALD "S"/3/IAS 54-20/Cotipora/CNT8	1987	
A	BRS 177	PF 83899/PF 813//F27141	1999	
A	BRS 179	BR35/PF 8596/3/PF 772003*2/PF 813//PF 83899	1999	
A	BRS 208	CPAC 89118/3/BR23//CEP19/PF85490	2001	x
A	BRS 220	Embrapa 16 TB 108	2003	x

Collection	Cultivar	Pedigree	Launch year	In recommendation to grow in 2022 [1]
A	BRS 254	Embrapa 22*3/ANA 75	2005	x
A	BRS 264	Buck Buck/Chiroca//Tui	2005	x
A	BRS 276	Embrapa 27*3/Klein H3247 a 33400//PF 93218	2008	
A	BRS 296	PF 93232/Cook*4/VPM1	2009	
AB	BRS 327	CEP 24/BRS 194	2010	x
AB	BRS 328	Klein H3394 a 3110/PF 990744	2012	
AB	BRS 331	PF 990606/WT 98109	2012	
AB	BRS 374	PF 88618/Koker 80.33//Frontana/Karl	2012	x
A	BRS Albatroz	PF 940301/PF 940395	2011	
A	BRS Buriti	Embrapa 27/Klein Orion	2003	
A	BRS Camboatá	PF 93232 Sel 14	2003	
B	BRS Gaivota	PF 940301/PF 940395	2011	x
AB	BRS Gralha Azul	BRS 209//Camboata/Lr37 (Lr37 = Cook*4/VPM1)	2012	x
A	BRS Guabiju	PF 86743/BR 23	2003	x
A	BRS Guamirim	EMB 27/Buck Nandu//PF93159	2005	x
A	BRS Louro	PF 869114/BR 23	2003	x
AB	BRS Pardela	BR 18/PF 9099	2007	x
A	BRS Parrudo	WT89109/TB0001	2012	x
B	BRS Sabiá	BRS 210/PF 980583	2014	x
A	BRS Tangará	BR 23*2/PF 940382	2007	x
A	BRS Timbaúva	BR 32/PF 869120	2003	

**Collection**	**Cultivar**	**Pedigree**	**Launch year**	**In recommendation to grow in 2022** [1]
AB	Campeiro	ORL 97217//BRS 177/Avante	2009	x
A	CD 104	PFAU "S"/IAPAR 17	1999	
A	CD 105	PFAU "S"/2*OCEPAR 14//IAPAR 41	1999	
A	CD 108	TAM200/Turaco	2003	
B	CD 1104	CD 108/BRS 220	2014	x
A	CD 113	Embrapa 27/OC 946	2004	
A	CD 114	PF 89232/OC 938	2004	
A	CD 115	OC 926/OC 935	2005	
A	CD 116	Milan/Munia	2006	
A	CD 117	PF 87373/OC 938	2007	
A	CD 118	Veery/Koel//Siren/3/Arivechi M 92	2008	
A	CD 119	BRS 49/CDI 0303	2009	
A	CD 121	ORL 95688/CD 116	2010	
A	CD 122	IPR 85/WT 96168	2010	
A	CD 123	BRS 177/CD 108	2010	
A	CD 150	CD 104/CD 108	2009	x
A	CD 151	BRS 120/ORL 95282	2012	
A	CD 154	CD 104/CDI 200104	2012	
B	CD 1550	Ônix/CDFAPA 2001129	2012	
B	Celebra	Marfim/Quartzo//Marfim	2014	x
A	Embrapa 22	VEE "S"/3/KLTO "S"/PAT 19//MO/JUP	1993	
A	Embrapa 42	LAP 689/MS 7936	1995	

Collection	Cultivar	Pedigree	Launch year	In recommendation to grow in 2022 [1]
B	FPS Nitron	ORL 94300/Ônix	2011	
A	Fundacep 300	BR 32/CEP 21//Ciano 79	2009	
A	Fundacep 51	CEP 88132/PG 876//BR 34/CRDN	2005	
A	Fundacep 52	CEP 88132/PG 876//BR 34/CRDN	2005	
A	Fundacep Bravo	Rubi/Fundacep 37	2010	
A	Fundacep Campo Real	CEP 889171/PF 869114//OR 1	2009	
AB	Fundacep Cristalino	BR 35/CEP 9291/4/BR 32/3/CNO 79/PF 70354/MUS "S"	2006	
A	Fundacep Horizonte	BRS 119/CEP 97184	2009	
A	Fundacep Nova Era	CEP 88132/PG 876//BR 34/CRDN	2004	
A	Fundacep Raizes	EMB 27/CEP 24/3/BUC "S"/FCT "S"//PF85229	2006	
A	IPR 128	VEE/LIRA//BOW/3/BCN/4/KAUZ	2006	
A	IPR 130	Rayon//VEE#6/TRAP#1	2007	
A	IPR 136	TAW/SARA//BAU/3/ND674*2/IAPAR 29	2007	
A	IPR 144	SERI*3/BUC/5/BOW/3/CAR 853/COC//VEE/4/OC 22	2009	x
AB	IPR 85	IAPAR 30/BR 18	1999	x
AB	IPR Catuara	LD 975/IPR 85	2012	x
B	Jadeíte 11	Campo Real/Vanguarda//Ônix	2012	
B	LG Oro	Fundacep 30/Fundacep Cristalino	2014	x
B	LG Prisma	BRS Timbaúva/Abalone	2014	x
AB	Marfim	ORL 94101/2*ORL 95688	2007	x
AB	Mirante	Ônix/Taurum//Ônix	2008	

Collection	Cultivar	Pedigree	Launch year	In recommendation to grow in 2022 [1]
A	Ônix	CEP 24/RUBI "S"	2002	
A	OR 1	Embrapa 27/Bagula "S"	1996	
B	ORS 1401	Abalone//ORL 99075/Ônix	2015	x
B	ORS 1403	INIA Tijereta/Alcover//Abalone	2016	x
B	ORS 1405	Quartzo/3/Fundacep 30/Ônix//Pampeano/4/Quartzo	2016	x
B	ORS Vintecinco	Vanguarda/Temu 2624-00	2013	x
A	Pampeano	ORL91274/ORL93807//ORL95711'S'	2004	
AB	Quartzo	Ônix/Avante	2007	x
AB	Safira	PF 9099/OR 1//Granito	2004	
AB	Supera	PF 9099/OR 1	2004	
B	Tbio Bandeirantes	IBIO 00718/Cronox//Alcover	2012	
B	Tbio Iguaçu	Quartzo/Safira	2012	x
B	Tbio Itaipu	Quartzo/Safira	2012	
B	Tbio Mestre	IBIO 00810/Cronox//ORL 002255	2012	x
B	Tbio Noble	Quartzo/ORL 97061//Marfim	2013	x
B	Tbio Pioneiro	Cronox/Vaqueano	2010	
B	Tbio Seleto	ORL 04300/Ônix	2012	
B	Tbio Sintonia	Marfim/Quartzo//Marfim	2013	x
B	Tbio Sinuelo	Quartzo/3/Fundacep 30/Ônix//Pampeano/4/Quartzo	2012	x
B	Tbio Tibagi	Supera/Ônix	2010	x
B	Tbio Toruk	Mirante/IBIO 0901//Quartzo	2014	x
B	TEC Frontale	ORL 95688/Embrapa 16	2012	

Collection	Cultivar	Pedigree	Launch year	In recommendation to grow in 2022 [1]
A	TEC Veloce	ORL 91256/FUNDACEP 29//BRS 177	2012	
A	TEC Vigore	Fundacep Cristalino/Pampeano	2012	
AB	Topazio	Pampeano "S"/Abalone	2011	
A	Turqueza	Pampeano/ORL 98231//Cronox	2011	
A	Valente	BR 18/Alcover	2004	
A	Vaqueano	IOR 951/ORL 957//Granito	2008	

[1] According to the “Informações Técnicas para Trigo e Triticale” (‘Informações técnicas para trigo e triticale - Safra 2022’, 2022).
