# Supplementary material for: Characterization of Brazilian spring wheat germplasm and its potential for increasing wheat genetic diversity in Canada
Source: Front Genet. 2023 Mar 17;14:1125940. doi: 10.3389/fgene.2023.1125940 (PMC10063806; doi:10.3389/fgene.2023.1125940)
Supplement: Supplementary file 2 [file DataSheet1.pdf]

ADON	acetyl-deoxynivalenol
APR	adult plant resistance
Bt	common bunt gene
CA	Canada
CAPS	cleaved amplified polymorphic sequence marker
Collection 'A'	Collection ‘A’ was composed of cultivars registered in Brazil from 1986 to 2012
Collection 'AB'	Collection 'AB': sixteren cultivars presented in collection A and collection B, old cultivars that were still being cultivated.
Collection 'B'	collection ‘B’ was composed by cultivars dated from 1999 to 2016
CV	coefficient of variation
DI	disease incidence
DNA	deoxyribonucleic acid
dNTP	Deoxynucleoside triphosphate
DON	deoxynivalenol
DS	disease severity
EMBRAPA	Brazilian Agricultural Research Corporation (Empresa Brasileira de Pesquisa Agropecuária)
Fg	*Fusarium graminearum*
FHB	Fusarium head blight
FHBInc	Fusarium head blight incidence
FHBInd	Fusarium head blight index
FHBSev	Fusarium head blight severity
hetero	heterozygosity
IT	infection type
KASP	competitive allele-specific PCR marker
Lr	leaf rust resistance genes
LSD	least significant difference
Ltn	leaf tip necrosis
MAS	marker-assisted selection
MR	moderately resistant
MS	moderately susceptible
neg	negative
NIV	nivalenol (Fusarium mycotoxin)
NSD	not sufficient data
NX-2	Fusarium mycotoxin, the structure of the molecule is identical to that of 3ADON except for the absence of a keto group at the 8 position carbon
p	probability
PCR	polymerase chain reaction
Pgt	*Puccinia graminis* Pers. f. sp. *tritici*
PltHeight	Plant height
Pm	powdery mildew gene
pos	positive
PPC3	Plant Pest Containment Level 3
Pst	*Puccinia striiformis* f. sp. *tritici*
QTL	quantitative trait loci
R	resistant
Rht	gibberellin-insensitive reduced height or sem-dwarf genes

S	susceptible
Sr	stem rust resistance genes
SSR	simple sequence repeats marker or microsatellites
STS	sequence-tagged sites
TKW	thousand kernel weight
TW	test weight
VRI	FHB visual rating index
Yr	yellow rust (stripe rust) gene
